# Supplementary material for: Noise-aware training of neuromorphic dynamic device networks
Source: Nat Commun. 2025 Oct 16;16:9192. doi: 10.1038/s41467-025-64232-1 (PMC12533029; doi:10.1038/s41467-025-64232-1)
Supplement: Supplementary file 1 — Supplementary Information [file 41467_2025_64232_MOESM1_ESM.pdf]

Supplementary Information for 'Noise-Aware Training of Neuromorphic Dynamic Device Networks'

1 Results of NSDE Modelling

Two dynamical systems with well-defined analytical descriptions—the nonlinear leaky integrator and the Duffing oscillator—were used as control cases to test the model fitting process shown in Figure 1. These systems provided 'ground-truth' data, allowing for precise control over timescales and noise magnitudes, and enabling direct calculation of gradients with respect to input and node dynamics. This capability is crucial for assesing the model's accuracy in approximating the gradients required for optimization. In contrast, these properties cannot be explicitly evaluated in physical systems due to the absence of differentiable mathematical descriptions and presence of experimental noise in measurements of state. Detailed definitions of the simulated systems' dynamics, along with figures demonstrating the neural-SDE model's high accuracy in capturing their behaviors, are available in Supplementary Figures 1, 3, 6, and 7.

1.1 Challenges in computing with physical dynamics

From the standpoint of each device within the network, the optimization of the external signal necessitates a consideration of its dynamic nature. Achieving a mathematically precise optimization process entails unravelling the system dependencies backwards in time, a procedure known as backpropagation through time (BPTT[1]). It is worth noting that any algorithm, including recent approximations of BPTT[2,3], designed to execute such optimization in the context of neural networks, assumes a certain mathematical understanding of how a system at time t depends on its past states. In mathematical terms, defining the i-th system dynamics and the external signal at time t through the variables $\mathbf{y}_i(t)$ and $\mathbf{s}_i(t)$ respectively, input optimization necessitates knowledge of the total derivatives $\frac{\mathrm{d}\mathbf{y}_i(t)}{\mathrm{d}\mathbf{s}_i(t')}$ for any $t' < t$. BPTT and its variants untangle these total derivatives through a chain rule involving the partial derivatives $\frac{\partial \mathbf{y}_i(t)}{\partial \mathbf{y}_i(t-\delta t)}$ and $\frac{\partial \mathbf{y}_i(t)}{\partial \mathbf{s}(t)}$ for all t in the considered temporal interval. Therefore, estimating these factors is crucial for training the network of interacting devices in a general setting.

Another challenging aspect in optimising interactions between physically defined dynamic systems lies in their intrinsic stochastic behaviours. Stochasticity can significantly impact the performance of networks, a challenge that becomes more apparent in dynamic systems where current noise realizations (or even stochasticity in the initial conditions) may abruptly influence future system behaviour. Consequently, we will adopt and formulate models that can also capture the stochasticity of the devices considered and provide an estimate of the uncertainty associated with a particular interaction.

The formulated neural-SDE

The framework outlined here leverages variants of neural ordinary/stochastic differential equations as differentiable models to capture the dynamics and stochasticity of devices. A Neural-ODE/SDE model was used to simulate a type of device, then a network of Neural-ODE/SDEs was adopted to simulate a network of interacting devices. The differentiation through the simulated system provided estimates of the necessary components to perform BPTT and to train the simulated interactions. The optimized parameters will be then transferred to the physically defined systems, where the performance is assessed. The following paragraph outlines the digital twins and introduces the required formalism.

Let us consider a dynamic system and the collection of information over experimentally measurable variables, denoted as $\mathbf{x}(t)$, representing the evolution of pertinent properties of the system under the influence of an external signal $\mathbf{s}(t)$.

In the initial phase, trajectories of responses $\left(\ldots,\mathbf{x}(t),\mathbf{x}(t+\delta t),\ldots\right)$ from the studied device are recorded for various input sequences $\left(\ldots,\mathbf{s}(t),\mathbf{s}(t+\delta t),\ldots\right)$. The range and statistics of these input sequences are deliberately defined to gather a comprehensive dataset of input/output responses. This dataset of trajectories is then employed to optimize the N-DE models, whose activities, which we denote as $\tilde{\mathbf{x}}(t)$, are trained to reproduce the corresponding real variables $\mathbf{x}(t)$.

Given the feed-forward structure of N-DEs in predicting the "next" system activity and the resulting Markovian prerequisites for their input representation, we enhance their input by incorporating delayed observations, as in prior works[4] on neural-ODEs. We define this augmented state at time t as $\tilde{\mathbf{y}}(t) = \left(\tilde{\mathbf{x}}(t),\tilde{\mathbf{x}}(t-\delta t),\ldots,\tilde{\mathbf{x}}(t-\mathrm{N}_{\mathrm{delay}}\delta t)\right)$, and the corresponding real variable as $\mathbf{y}(t) = \left(\mathbf{x}(t),\mathbf{x}(t-\delta t),\ldots,\mathbf{x}(t-\mathrm{N}_{\mathrm{delay}}\delta t)\right)$. While neural-ODEs can replicate deterministic dynamics[4,5] and are traditionally trained with cost functions as mean-squared error, neural-SDE can additionally capture device stochastic behaviour[6].

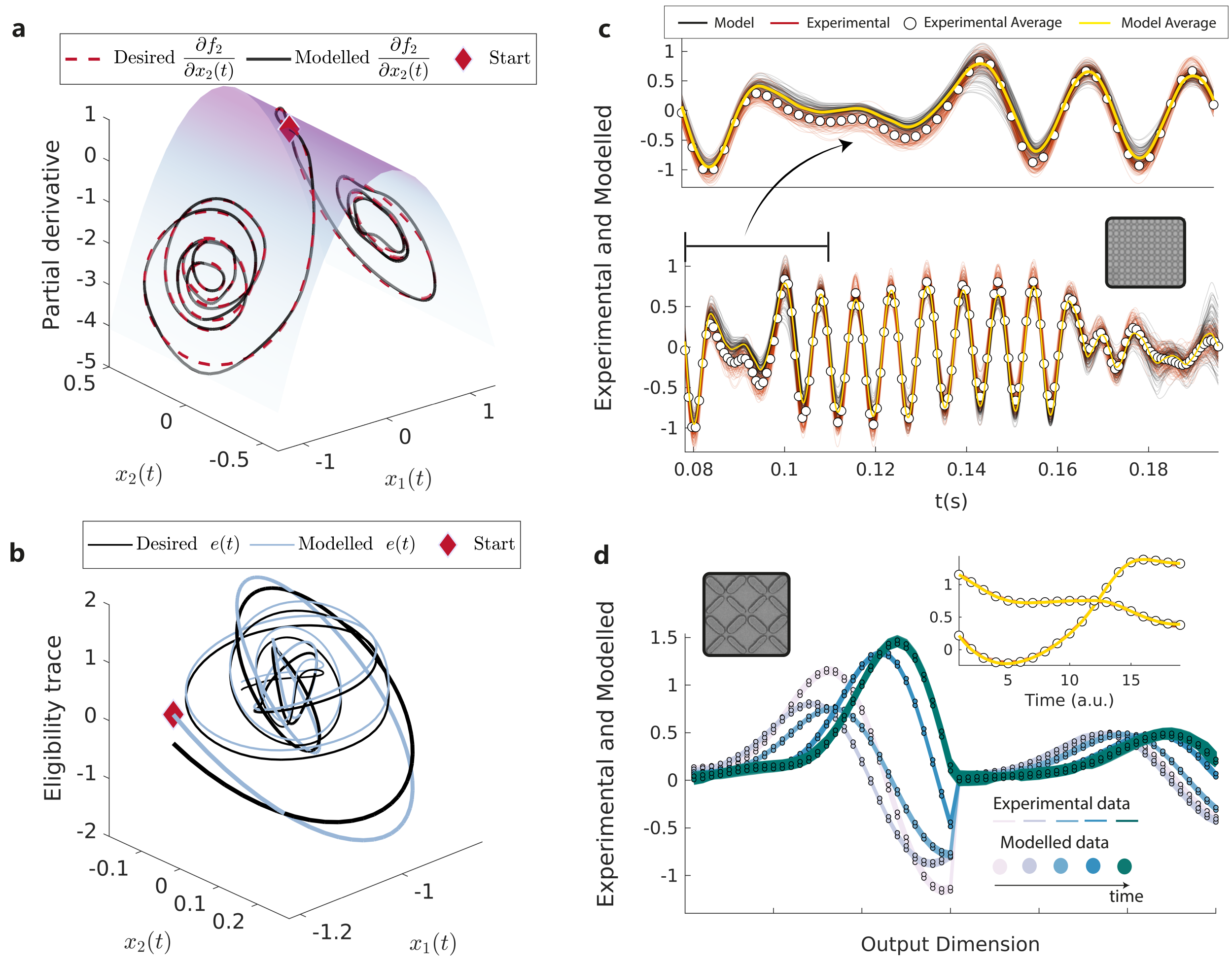

Supplementary Figure 1. Modelling of simulated and experimental dynamical systems. Panel **a** illustrates a simulated example of a partial derivative of the acceleration (f_2) with respect to position for the Duffing oscillator. The surface represents the partial derivative as position ($x_1(t)$) and velocity ($x_2(t)$) of the system vary. The example trajectories compare the gradient over time, calculated both analytically (red) and via differentiation of the Neural-SDE model (black), for two input sequences, showing excellent agreement. Panel **b** provides a more general view of the model's ability to act as a surrogate for device gradients; here, we adopt eligibility traces that accumulate gradient information (see Main text and Supplementary Information for more details). The difference between the desired and modelled eligibility traces increases due to error accumulation. Panel **c** compares responses generated via the Neural-SDE model (black and yellow lines) and experimentally gathered data of the NRA device (red lines, white circles) for 100 repetitions of a random input sequence. Panel **d** illustrates the Neural-SDE's ability to model the high-dimensional, experimentally measured responses of an artificial spin-vortex-ices (ASVI) device. Here, the x-axis corresponds to the different output dimensions of the device responses, while the colours reflect the temporal evolution. Even for this multivariate system, the model (coloured lines) accurately captures the system behaviour (dots).

Neural-ODE and SDE models

This section is dedicated to providing more details on the procedure adopted in training Neural-ODE and SDEs. Let us consider a dynamic system described by an N_X-dimensional observable variable $\mathbf{x}(t)$ and driven by an N_S-dimensional external signal $\mathbf{s}(t)$. Using $\mathbf{y}(t)$ as an augmented version of the dynamic of $\mathbf{x}(t)$ aimed to capture higher order moments and to provide a Markovian representation of the evolution of the system, we can define a dataset of transitions $\mathscr{D} = \left\{ \dots, \left(\mathbf{y}(t-\delta t), \mathbf{s}(t), \mathbf{y}(t) \right), \dots \right\}$. The value of N_{delay} specifies the number of delayed activities concatenated in the vector $\mathbf{y}$, which consequently $\in \mathscr{R}^{N_X \times (N_{delay}+1)}$.

Considering the evolution of a variable $\mathbf{y}(t)$ across time, a neural-ODE corresponds to an ordinary differential equation parametrized via a neural network of the form

$$d\tilde{\mathbf{y}}(t) = \mathbf{f}\left(\tilde{\mathbf{y}}(t), \mathbf{s}(t), t | \phi^f\right) dt \quad (1)$$

where ϕ^f are the parameters of the network, the tilde $(\tilde{\cdot})$ is introduced to distinguish the target from the generated variables, and $\mathbf{s}(t)$ is an external signal. As such, given an initial condition $\mathbf{y}(t_0)$ and a driving signal $\mathbf{s}(t)$, the neural-ODE will generate trajectories $\tilde{\mathbf{y}}(t)$ through iterative numerical integration. The input to the neural network $\mathbf{f}$ is thus a vector containing $\tilde{\mathbf{y}}$, $\mathbf{s}$ and a measure of time (which can also be included in the signal $\mathbf{s}(t)$). Neglecting the latter temporal information, the dimensionality of the input to the $\mathbf{f}$ network is $\mathrm{N_X} \times (\mathrm{N}_{delay} + 1) + \mathrm{N_S}$. For a better exposition, it is now useful to expand the above equation into its different delayed components

$$\begin{pmatrix} \mathrm{d}\tilde{\mathbf{x}}(t) \\ \mathrm{d}\tilde{\mathbf{x}}(t-\delta t) \\ \vdots \end{pmatrix} = \begin{pmatrix} \mathbf{f}_1(\tilde{\mathbf{y}}(t), \mathbf{s}(t), t|\phi^f) \\ \mathbf{f}_2(\tilde{\mathbf{y}}(t), \mathbf{s}(t), t|\phi^f) \\ \vdots \end{pmatrix} \mathrm{d}t \tag{2}$$

where we used the term ϕ^f for the different parameters defining $\mathbf{f}_1, \mathbf{f}_2, ...$ for simplicity of notation. To be more precise, the parameters ϕ^f are shared among the $\mathbf{f}_i$ functions but for the different read-outs, which lead to the various $\mathbf{f}_i$ in the output layer of the network. This notation is also adopted in other equations defining the neural-SDE models. We observe how, given that optimization of $\tilde{\mathbf{y}}(t)$ should lead such a variable to contain delayed system activities, it would be possible to impose a solution to $\mathbf{f}_2, ..., \mathbf{f}_{\mathrm{N}_{delay}+1}$ a priori. An example of this would correspond to set $\mathbf{f}_2 = \dfrac{\tilde{\mathbf{x}}(t) - \tilde{\mathbf{x}}(t-\delta t)}{\delta t}$, or in other words to exploit our knowledge that the evolution of $\tilde{\mathbf{x}}(t-\delta t)$ is $\tilde{\mathbf{x}}(t)$, which the model has already computed. In practice, we find that such an imposition is not necessary and that allowing training of $\mathbf{f}_2, ..., \mathbf{f}_{\mathrm{N}_{delay}+1}$ can marginally improve performance while the system will naturally discover to act as a shift register for the delayed variables.

Considering now the above-defined dataset $\mathscr{D}$, we can sample segments of trajectories with random starting times t_0 to define a batch of sequences used as targets for the model. The stochasticity in the selection of the starting times permits the batch sequence dynamics to be uncorrelated, and it is particularly advised for long temporal dynamics. For each sequence, the neural-ODE is initialized at the values $\tilde{\mathbf{y}}(t_0) = \mathbf{y}(t_0)$ and will generate a response $\tilde{\mathbf{y}}(t)$ for the intervals considered. Optimization of the parameters ϕ^f is achieved through the minimization of a cost function that reflects the discrepancy between the target and the generated sequences. Defining $\delta_j(t) = \tilde{\mathbf{y}}_j(t) - \mathbf{y}_j(t)$, the mean-squared error function to be minimized for a specific sequence is

$$\mathscr{L}_{\phi^f} = \sum_t \sum_j \delta_j(t)^2 \tag{3}$$

where the $t \in [t_0 \; t_0 + \mathrm{T}]$ is the interval considered and T defines the temporal length of the segment of the sequence in question. In practice, the error function will be defined and averaged across all segments of the considered minibatch to iteratively update ϕ^f.

While the neural-ODE model is effective at capturing deterministic dynamics, a neural-SDE aims to additionally capture the stochastic behaviour of the system in consideration. It is defined as

$$\mathrm{d}\tilde{\mathbf{y}}(t) = \mathbf{f}\big(\tilde{\mathbf{y}}(t), \mathbf{s}(t), t|\phi^f\big)\mathrm{d}t + \mathbf{g}\big(\tilde{\mathbf{y}}(t), \mathbf{s}(t), t|\phi^g\big)\mathrm{d}\mathbf{W} \tag{4}$$

where $\mathrm{d}\mathbf{W}$ is the derivative of the Wiener process, and ϕ^g are the parameters related to the stochastic component. This model is thus parametrized by two neural networks $\mathbf{f}$ and $\mathbf{g}$, whose inputs are the $\mathrm{N_X} \times (\mathrm{N}_{delay} + 1) + \mathrm{N_S}$ dimensional concatenation of $\tilde{\mathbf{y}}$ and $\tilde{\mathbf{s}}$. In this case, we generally found that it is important to constrain the function $\mathbf{g}$ to generate stochasticity only on the more recent $\mathrm{N_X}$-dimensional vector $\mathbf{x}(t)$, mathematically leading to

$$\begin{pmatrix} \mathrm{d}\tilde{\mathbf{x}}(t) \\ \mathrm{d}\tilde{\mathbf{x}}(t-\delta t) \\ \vdots \\ \mathrm{d}\tilde{\mathbf{x}}(t-\mathrm{N_{delay}}\delta t) \end{pmatrix} = \begin{pmatrix} \mathbf{f}_1(\tilde{\mathbf{y}}(t), \mathbf{s}(t), t|\phi^f) \\ \mathbf{f}_2(\tilde{\mathbf{y}}(t), \mathbf{s}(t), t|\phi^f) \\ \vdots \\ \mathbf{f}_{\mathrm{N}_{delay}+1}(\tilde{\mathbf{y}}(t), \mathbf{s}(t), t|\phi^f) \end{pmatrix} \mathrm{d}t + \begin{pmatrix} \mathbf{g}_1(\tilde{\mathbf{y}}(t), \mathbf{s}(t), t|\phi^g) \\ \mathbf{0} \\ \vdots \\ \mathbf{0} \end{pmatrix} \mathrm{d}\mathbf{W} \tag{5}$$

The reason for this lies in the additive nature of the noise and can be understood by envisioning the flow of information in the system through the delays. As the predicted activity $\tilde{\mathbf{x}}(t+\delta t)$ at time t shifts through the $\mathbf{f}_i$ functions to $\tilde{\mathbf{x}}(t-\delta t)$ at time $t+\delta t$, $\tilde{\mathbf{x}}(t-2\delta t)$ at time $t+2\delta t$ and so on, the stochastic function should not additionally contribute to value of such a variable multiple times.

Having defined the basic neural-SDE model, we leave the details of the learning algorithm and of the use of auxiliary variables to the next section.

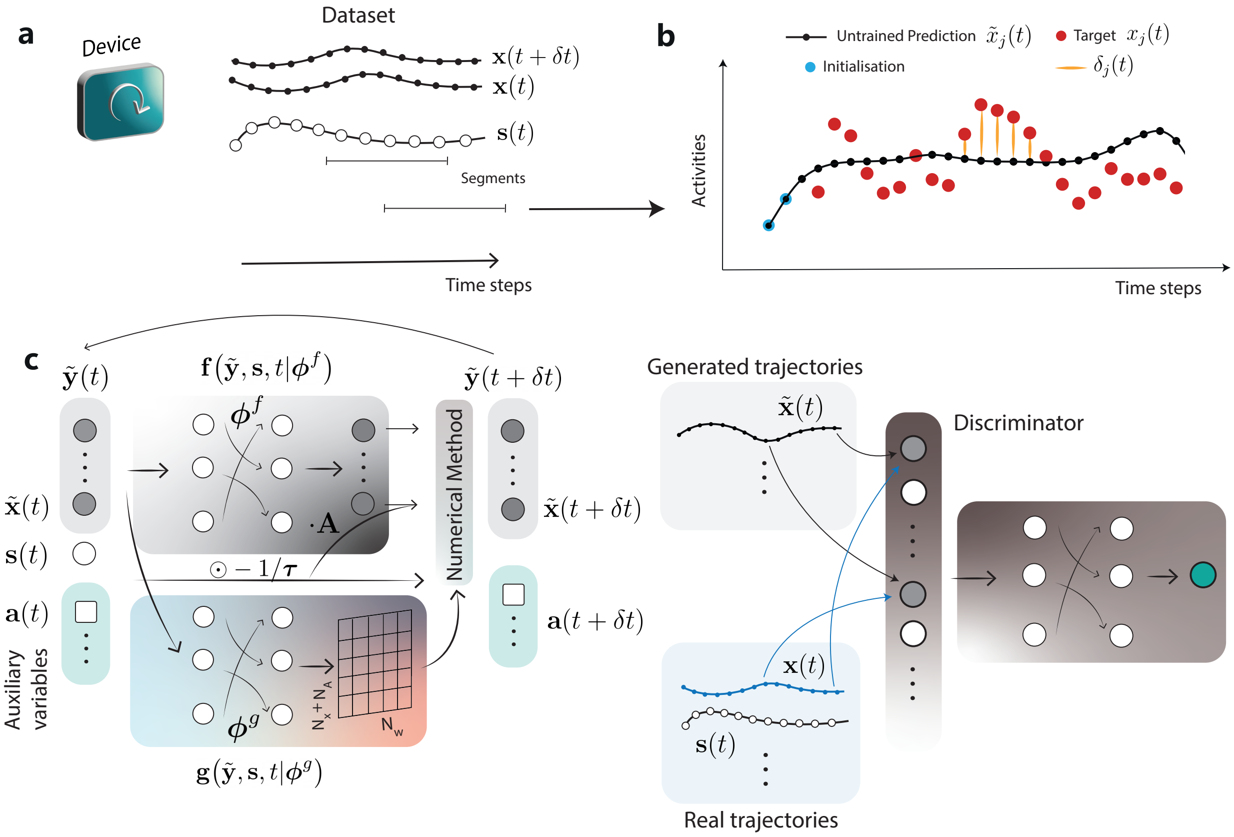

Supplementary Figure 2. **a** A scheme of the process of creating a dataset used to fit an N-DE model. The input data for initial conditions are the concatenation of external input at time t_0, $s(t_0)$, and a history buffer of experimentally measured states, $\mathbf{x}(t_0)$, are the initial conditions of the N-DE model, which is asked to produce trajectories that mimic the measured experimental output for the next increment in time, $\mathbf{x}(t+\delta t)$. The optimization process is depicted in panel **b** for an example segment of trajectories. The initial conditions are depicted in blue and correspond to the starting activities of the N-DE, whose generated response is reported in black. The optimization process of a neural-ODE typically involves minimization of the mean-squared error of $\delta(t)$, which is simply the difference between the generated and target activities, over the mini-batch of segments considered. Panel **c** shows the network architecture for the SDE model. A neural network **f** takes a concatenation of inputs $s(t)$ and a history buffer of past states $\tilde{\mathbf{x}}(t)$ as input, where the first entry of vector $\tilde{\mathbf{x}}$ is the device activity at time t, $\tilde{y}(t)$. A seperate network is used to simulate the stochastic response, denoted by **g**, with a set of auxilliary variables $\mathbf{a}(t)$ concatenated to the input for **f** used to generate noise of variable timescales. The output from both networks feed into a stochastic numerical integration scheme denoted as the Numerical Method. This produces predictions for the inputs to the next timestep $\tilde{\mathbf{x}}(t+\delta t)$, where the first entry serves as prediction for model device output at the next timestep, $\tilde{y}(t+\delta t)$, as well as an update for the auxilliary variables $a(t+\delta t)$. The prediction is taken, and outputs are fed back recursively as input for the next timestep, shown by the arrow. Neural network **g** is trained with a generative-adversarial network approach, where a discriminator network (shown on the right) is used to determine whether generated trajectories $\tilde{\mathbf{x}}(t)$ belong to the distribution of experimentally gathered trajectories $\mathbf{x}(t)$. The model is optimized until the generator network produces outputs that the discriminator cannot distinguish from the true distribution.

Neural-SDE optimization and coloured noise

We notice how in the above definition of the neural-SDE (Eq.5) the stochastic realizations of $\mathbf{g}_1 \mathrm{d}\mathbf{W}$ are integrated through the temporal kernel defined by $\mathbf{f}_1$. This leads the model to exhibit a limited ensemble of autocorrelation structures and its inability to capture coloured noise. To circumvent this limitation, we augmented the neural-SDE representation through auxiliary variables $\mathbf{a}(t)$ that operate over a wide range of timescales. Augmentation of stochastic differential equations with Ornstein-Uhlenbeck process is a commonly adopted strategy to introduce richer noise characteristics. The adapted neural-SDE equations, already

expanded for the different delayed inputs, are

$$\begin{pmatrix} \mathrm{d}\tilde{\mathbf{x}}(t) \\ \mathrm{d}\tilde{\mathbf{x}}(t-\delta t) \\ \vdots \\ \mathrm{d}\tilde{\mathbf{x}}(t-\mathrm{N}_{\mathrm{delay}}\delta t) \\ \mathrm{d}\mathbf{a}(t) \end{pmatrix} = \begin{pmatrix} \mathbf{f}_1(\tilde{\mathbf{y}}(t),\mathbf{s}(t),t|\phi^f) + \mathbf{A}\mathbf{a}(t) \\ \mathbf{f}_2(\tilde{\mathbf{y}}(t),\mathbf{s}(t),t|\phi^f) \\ \vdots \\ \mathbf{f}_{\mathrm{N}_{\mathrm{delay}}+1}(\tilde{\mathbf{y}}(t),\mathbf{s}(t),t|\phi^f) \\ -\boldsymbol{\tau}^{-1}\mathbf{a}(t) \end{pmatrix} \mathrm{d}t + \begin{pmatrix} \mathbf{g}_1(\tilde{\mathbf{y}}(t),\mathbf{s}(t),t|\phi^g) \\ \mathbf{0} \\ \vdots \\ \mathbf{0} \\ \mathbf{g}_a(\tilde{\mathbf{y}}(t),\mathbf{s}(t),t|\phi^g) \end{pmatrix} \mathrm{d}\mathbf{W} \tag{6}$$

where $\mathbf{a}$ are the N_a-dimensional auxiliary variables, $\boldsymbol{\tau}$ is the $\mathrm{N}_a \times \mathrm{N}_a$-dimensional diagonal matrix defining the different timescales, $\mathbf{A}$ is a trainable $\mathrm{N_X} \times \mathrm{N_a}$ connectivity matrix that links the auxiliary variables to the current activities $\mathbf{x}(t)$. As before, we use the same notation for the parameters ϕ^g of the different terms $\mathbf{g}_1$ and $\mathbf{g}_a$ for simplicity of notation. We indicate as $\mathbf{g}$ the overall function (in square brackets) that is multiplied by the Wiener process. A scheme of the corresponding network architecture is given in Figure 2 c, depicting the networks $\mathbf{f}(\tilde{\mathbf{y}}(t),\mathbf{s}(t),t|\phi^f) : \mathbb{R}^{\mathrm{N_X}(\mathrm{N}_{delay}+1)} \to \mathbb{R}^{\mathrm{N_X}(\mathrm{N}_{delay}+1)}$, $\mathbf{g}(\tilde{\mathbf{y}}(t),\mathbf{s}(t),t|\phi^g) : \mathbb{R}^{\mathrm{N_X}(\mathrm{N}_{delay}+1)} \to \mathbb{R}^{(\mathrm{N_X}+\mathrm{N_a})\times \mathrm{N_W}}$, where we excluded the zero elements in the dimensionality of its output, and how they interact.

Optimization of the neural-SDE is accomplished through a generative adversarial network paradigm where the neural-SDE is treated as a generator similarly to previous works[6] and a discriminator provides the feedback for optimization. In our settings, the discriminator is a feedforward network, in Figure 2(c), an MLP, receiving trajectories of dynamics and the corresponding driving signals as inputs. Denoting with $\mathbf{s}_d$ the input to the discriminator network, $\mathbf{s}_d^g = \Big(\mathbf{s}(t_0), \tilde{\mathbf{x}}(t_0), \mathbf{s}(t_0+\delta t), \tilde{\mathbf{x}}(t_0+\delta t), ..., \mathbf{s}(t_0+\mathrm{T}), \tilde{\mathbf{x}}(t_0+\mathrm{T})\Big)$ for the generated activities and $\mathbf{s}_d^r = \Big(\mathbf{s}(t_0), \mathbf{x}(t_0), \mathbf{s}(t_0+\delta t), \mathbf{x}(t_0+\delta t), ..., \mathbf{s}(t_0+\mathrm{T}), \mathbf{x}(t_0+\mathrm{T})\Big)$ for the "real" trajectories, where we recall that T is the temporal length of the trajectory considered. Thus, the discriminator is a network $\mathbf{d} : \mathbb{R}^{(\mathrm{N_X}+\mathrm{N_S})(T+1)} \to \mathbb{R}$, whose output reflects a confidence estimate that the input data has not been generated by the neural-SDE. The optimization is achieved via a Wasserstein loss function with gradient penalty[7], which for completeness is given by

$$\underset{\mathbf{s}^g}{\mathbb{E}}\{d(\mathbf{s}_d^g)\} - \underset{\mathbf{s}^r}{\mathbb{E}}\{d(\mathbf{s}_d^r)\} + (||\underset{\hat{\mathbf{s}}}{\nabla} d(\hat{\mathbf{s}}_d)||_2 - 1)^2, \tag{7}$$

where the $\hat{\mathbf{d}}$ corresponds to linearly interpolated data between $\mathbf{s}_d^r$ and $\mathbf{s}_d^g$[7]. The cost function for the generator is typically

$$-\underset{\mathbf{s}^g}{\mathbb{E}}\{d(\mathbf{s}_d^g)\} \tag{8}$$

However, we noticed that such a formulation can lead the training process to undesirable local minima because of mode collapses, i.e. the tendency of the network to only capture specific statistical properties of the data, or oscillations in the performance. Denoting with $\mathbf{d}^{(i)}$ the activities of the i-th layer of the discriminator, we reformulated the minimization process of the generator in a contrastive learning fashion with the new cost function

$$||\langle \mathbf{d}^{(i)}(\mathbf{s}^r)\rangle - \langle \mathbf{d}^{(i)}(\mathbf{s}^g)\rangle||_2^2 + ||\sigma\big(\mathbf{d}^{(i)}(\mathbf{s}^r)\big) - \sigma\big(\mathbf{d}^{(i)}(\mathbf{s}^g)\big)||_2^2 \tag{9}$$

where $\langle\cdot\rangle$ and $\sigma(\cdot)$ are the average and standard deviation computed over the minibatch considered. We observed that this formulation led to slower convergence time of the algorithm but to improved solutions. Finally, while the sets of parameters of a neural-SDE have been previously trained simultaneously via the discriminator, we found it necessary to improve our control over the solution. Optimization of GANs can indeed require intensive fine-tuning[8] because of non-converging behaviours and mode-collapse difficulties. To partially circumvent these problems, we pre-trained the deterministic component of the neural-SDE, i.e. the ODE part, with a mean-squared error function. In such a way, the network is already capable of capturing the average behaviour of the dynamics when we start the optimization through the discriminator. Of course, this can introduce a trade-off on when to stop the deterministic training for all those cases where noise can dramatically affect the average system evolution. For instance, if a dynamic system can exhibit bifurcations, it is challenging to optimize the deterministic component, which might interpolate among the different possible trajectories after bifurcation. However, we found that this apparent complexity can be circumvented by augmenting the input to the model including more delayed activities (increasing N_{delay}) and that pretraining the neural-ODE would still be less tedious than fine-tuning the parameters for end to end optimization of the neural-SDE through the discriminator. It is finally important to notice that optimization of the N-DE models has been achieved by backpropagating through the numerical method as in previous neural-SDE formulations[6].

In the context of capturing device dynamics, we also advise adopting statistical metrics computed on an appropriately prepared validation set to control and stop the optimization of the neural-SDE. In particular, we generated trajectories of responses of the considered device when subjected to repetitions of the same (approximately the same) external signal. We repeated then this process for multiple signals, and computed metrics evaluating the discrepancy between the average, variance, and autocovariance structure between the generated and physical device responses. We then chose the model across training that corresponds to the lowest error, computed simply by summing across the different metrics. The result of this procedure permitted a high degree of control over the solution.

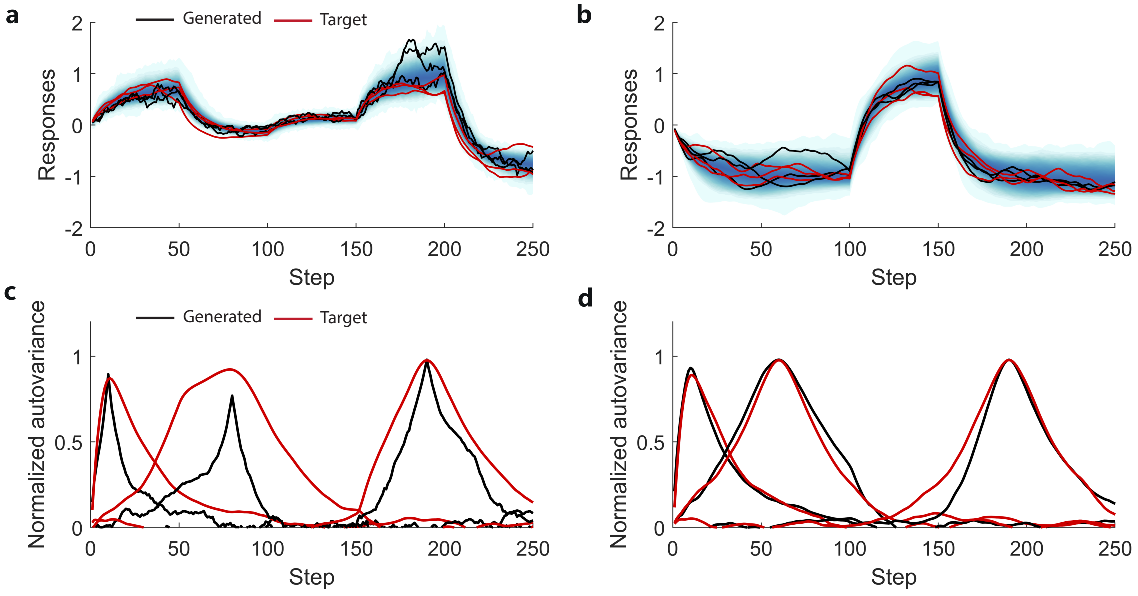

Supplementary Figure 3. The figure illustrates ability for neural-SDEs to capture autocorrelation structure of coloured noise. The dynamic system in question is a simple non-linear leaky integrator, where its stochasticity lies on a slower timescale in comparison to its leakage term, which defines its deterministic temporal kernel. Panels **a** and **c** (**b** and **d**) correspond to a neural-SDE without (with) augmentation of the auxiliary variables. **a** and **c** report examples of generated trajectories (black) compared to the corresponding system dynamics (red), where the blue area reflects the dispersion of the distribution of the dynamic process. Given that both models can capture the dispersion of the process, the dynamics generated by the neural-SDE of panel **a** exhibit rapidly changing stochastic behaviour, not capturing the smoother trends of the reference system. This translates into an inaccurate reproduction of the autocovariance structure, shown in panel **c**, where the different trends correspond to the diverse reference times over which the autocovariance function is computed. In contrast, the dynamics of the augmented neural-SDE are in considerably improved agreement with the process under study (panel **b** and **d**). Despite the simplicity of this example, the result shows how augmentation of the neural-SDE model can strongly improve its expressivity, and it might be necessary to capture stochastic behaviours that are only partially entangled to the temporal kernel of its deterministic component.

1.2 Optimization of a dynamic physical neural network

An optimized neural-SDE is used as a complex node of a network that simulates the ensemble of interacting devices.

Neural-SDE as nodes

The framework formulation assumes that the i-th device, considered in isolation, can be described as

$$\mathrm{d}\mathbf{y}_i(t) = \mathbf{f}_i\big(\mathbf{y}_i(t),\mathbf{s}_i(t),t\big)\mathrm{d}t + \mathbf{g}_i\big(\mathbf{y}_i(t),\mathbf{s}_i(t),t\big)\mathrm{d}\mathbf{W} \tag{10}$$

where the subscripts i on the $\mathbf{f}$ and $\mathbf{g}$ functions are introduced to distinguish between the different devices' behaviours explicitly. As before, the augmented variable $\mathbf{y}_i$ is defined as $\mathbf{y}_i(t) = \Big(\mathbf{x}_i(t),\mathbf{x}_i(t-\delta t),...,\mathbf{x}_i(t-\mathrm{N}_{\mathrm{delay}}\delta t)\Big)$, where $\mathbf{x}_i$ corresponds to the experimentally measurable time-dependent features.

In the general context of a physically implemented network, the variables $\mathbf{y}_i(t)$ adopted to capture the devices' dynamics might not correspond to the information exchanged by the different devices. We can instead safely assume that interactions occur via the variables $\mathbf{y}_i^{\pi} = \boldsymbol{\pi}(\mathbf{y}_i(t))$, where $\boldsymbol{\pi}$ is an arbitrary projection leading to a lower dimensional space. The consideration of a network of interacting systems implies that

$$\mathbf{s}_i(t) = \mathbf{h}_i\Big(\mathbf{y}_1^{\pi}(t),...,\mathbf{y}_{\mathrm{N}}^{\pi}(t),\mathbf{s}^{task}(t)|\boldsymbol{\theta}_i\Big) \tag{11}$$

where $\mathbf{s}^{task}(t)$ is a task-dependent input that drives the system, and $\mathbf{h}_i$ corresponds to the sub-network of nodes adjacent to the i-th device and that is parametrized by the connectivity $\boldsymbol{\theta}_i$. Depending on the specific network architecture, $\mathbf{h}_i$ might be a function of a subset of the above arguments. For instance, devices in the second layer of a typical feedforward structure will be connected only to the first layer of devices without receiving an external input $\mathbf{s}^{task}(t)$. We denote as $\boldsymbol{\theta}$ the complete set

of network parameters, while the system output is defined as a read-out $\mathbf{o}(t) = \mathbf{W}^{\mathrm{o}}\big(\mathbf{y}_1^{\pi}(t), ..., \mathbf{y}_{\mathrm{N}}^{\pi}(t)\big)$. Given an example error function $E(t) = \big(\mathbf{o}(t) - \mathbf{o}^{\mathrm{task}}(t)\big)^2$, where $\mathbf{o}^{\mathrm{task}}$ is a task-dependent target, minimization of $E(t)$ with respect to $\boldsymbol{\theta}$ leads to

$$\frac{\mathrm{d}E(t)}{\mathrm{d}\boldsymbol{\theta}} = \frac{\partial E(t)}{\partial \mathbf{o}(t)} \sum_i \frac{\partial \mathbf{o}(t)}{\partial \mathbf{y}_i^{\pi}(t)} \frac{\mathrm{d}\mathbf{y}_i^{\pi}(t)}{\mathrm{d}\boldsymbol{\theta}} \tag{12}$$

where $\frac{\mathrm{d}\mathbf{y}_i^{\pi}(t)}{\mathrm{d}\boldsymbol{\theta}}$ requires differentiation through the system's dynamics (Eq.10) and of the terms $\mathbf{h}_i$ corresponding to the network structure. As a consequence, Eq.12 is intractable for physical systems that lack a precise differentiable description.

A device i belongs to a type of devices, whereas type is a set D of devices that have common statistical properties and whose behavioural differences are caused by fabrication variability (D might correspond to different ASVIs, for instance). The neural-SDE for device i is

$$\mathrm{d}\tilde{\mathbf{y}}_i(t) = \mathbf{f}_d\big(\tilde{\mathbf{y}}_i(t), \mathbf{s}_i(t), t|\phi_d^f\big)\mathrm{d}t + \mathbf{g}_d\big(\tilde{\mathbf{y}}_i(t), \mathbf{s}_i(t), t|\phi_d^g\big)\mathrm{d}\mathbf{W} \tag{13}$$

where the subscript d indicates the presence of type-dependent functions and will be omitted for simplicity of notation. The external signal $\mathbf{s}_i(t)$ can be further augmented through a device identifier to account for fabrication variability, and we recall how the parameters of the neural-SDE are optimized to mimic the reference systems' behaviours as shown in the previous sections. The optimized neural-SDEs are then adopted to estimate the unknown terms of Eq.12 through

$$\frac{\mathrm{d}E(t)}{\mathrm{d}\boldsymbol{\theta}} \approx \frac{\partial E(t)}{\partial \tilde{\mathbf{o}}(t)} \sum_i \frac{\partial \tilde{\mathbf{o}}(t)}{\partial \tilde{\mathbf{y}}_i^{\pi}(t)} \frac{\mathrm{d}\tilde{\mathbf{y}}_i^{\pi}(t)}{\mathrm{d}\boldsymbol{\theta}} \tag{14}$$

While Eq.14 can be computed through auto-differentiation tools over the dynamics defined in Eq.13, it is still relevant to understand the mathematics behind the framework and expand the terms of Eq.12.

An isolated device and its eligibility trace

As a starting point and to introduce the eligibility traces of Figure 1(d) (Main text), consider an isolated device driven by a signal $\mathbf{s}^{task}(t)$ with activity $\mathbf{y}(t)$, whose total derivative can be unravelled in discrete time as

$$\frac{\mathrm{d}\mathbf{y}^{\pi}(t)}{\mathrm{d}\boldsymbol{\theta}} = \frac{\partial \mathbf{y}^{\pi}(t)}{\partial \mathbf{y}(t)} \left\{ \frac{\partial \mathbf{y}(t)}{\partial \boldsymbol{\theta}} + \frac{\partial \mathbf{y}(t)}{\partial \mathbf{y}(t-\delta t)} \left\{ \frac{\partial \mathbf{y}(t-\delta t)}{\partial \boldsymbol{\theta}} + \frac{\partial \mathbf{y}(t-\delta t)}{\partial \mathbf{y}(t-2\delta t)} \left[\frac{\partial \mathbf{y}(t-2\delta t)}{\partial \boldsymbol{\theta}} + ... \right] \right\} \right\} \tag{15}$$

which can be estimated through the neural-SDE dynamics adopting the approximation $\mathbf{y}(t) = \tilde{\mathbf{y}}(t)$ in Eq.15. Figure 4 shows how $\frac{\partial \tilde{\mathbf{y}}(t)}{\partial \boldsymbol{\theta}}$ and $\frac{\partial \tilde{\mathbf{y}}(t)}{\partial \tilde{\mathbf{y}}(t-\delta t)}$ are respectively computed differentiating the neural-SDE model, where the first term follows the backward path via the model's external input. If such terms are accurate estimators of the reference system regardless of the external input and previous activities, differentiation of the neural-SDE will be successful at optimising the connectivity $\boldsymbol{\theta}$. A comparison of the Jacobians $\frac{\partial \tilde{\mathbf{y}}(t)}{\partial \tilde{\mathbf{y}}(t-\delta t)}$ and $\frac{\partial \mathbf{y}(t)}{\partial \mathbf{y}(t-\delta t)}$ is shown in the Main text, Figure 1(c), for the Duffing oscillator.

Another more general approach to control the effectiveness of the model at capturing backward dependencies is to focus directly on the estimation of the total derivative $\frac{\mathrm{d}\mathbf{y}_i^{\pi}(t)}{\mathrm{d}\boldsymbol{\theta}}$. We can rewrite the terms of Eq.15 as

$$\frac{\partial \mathbf{y}(t')}{\partial \boldsymbol{\theta}} = \frac{\partial \mathbf{y}(t')}{\partial \mathbf{h}\big(\mathbf{s}^{task}(t')|\boldsymbol{\theta}\big)} \frac{\partial \mathbf{h}\big(\mathbf{s}^{task}(t')|\boldsymbol{\theta}\big)}{\partial \boldsymbol{\theta}} = \frac{\partial \mathbf{y}(t')}{\partial \mathbf{s}(t')} \mathbf{s}(t')\boldsymbol{\theta}^{-1} \tag{16}$$

in which we assume $\mathbf{s}(t') = \mathbf{s}^{task}(t')\boldsymbol{\theta}$. This implies that Eq.15 can be rewritten as

$$\frac{\mathrm{d}\mathbf{y}^{\pi}(t)}{\mathrm{d}\boldsymbol{\theta}} = \frac{\partial \mathbf{y}^{\pi}(t)}{\partial \mathbf{y}(t)} \left\{ \frac{\partial \mathbf{y}(t)}{\partial \mathbf{s}(t)} \mathbf{s}(t) + \frac{\partial \mathbf{y}(t)}{\partial \mathbf{y}(t-\delta t)} \left[\frac{\partial \mathbf{y}(t-\delta t)}{\partial \mathbf{s}(t-\delta t)} \mathbf{s}(t-\delta t) + ... \right] \right\} \boldsymbol{\theta}^{-1} = \frac{\partial \mathbf{y}^{\pi}(t)}{\partial \mathbf{y}(t)} \mathbf{e}(t)\boldsymbol{\theta}^{-1} \tag{17}$$

The variable $\mathbf{e}(t)$ is the eligibility trace and can be computed[2] through the recursive form $\mathbf{e}(t) = \frac{\partial \mathbf{y}(t)}{\mathbf{y}(t-\delta t)}\mathbf{e}(t-\delta t) + \frac{\partial \mathbf{y}(t)}{\partial \mathbf{s}(t)}\mathbf{s}(t)$, starting from $\mathbf{e}(t_0) = 0$. In the main text, we compare $\mathbf{e}(t)$ with $\tilde{\mathbf{e}}(t)$, which is estimated through the neural-SDE of dynamic $\tilde{\mathbf{y}}(t)$.

Interacting devices and backpropagation

We now expand Eq.12 considering the whole network of interacting devices. Starting from the i-th node, BPTT through the network involves the chain of derivatives

$$\frac{\mathrm{d}\mathbf{y}_i(t)}{\mathrm{d}\boldsymbol{\theta}} = \frac{\partial \mathbf{y}_i(t)}{\partial \boldsymbol{\theta}} + \sum_k \frac{\partial \mathbf{y}_i(t)}{\partial \mathbf{y}_k(t-\delta t)} \left\{ \frac{\partial \mathbf{y}_k(t-\delta t)}{\partial \boldsymbol{\theta}} + \sum_j \frac{\partial \mathbf{y}_k(t-\delta t)}{\partial \mathbf{y}_j(t-2\delta t)} \left[\frac{\partial \mathbf{y}_j(t-2\delta t)}{\partial \boldsymbol{\theta}} + \ldots \right] \right\} \tag{18}$$

where, for instance, the k elements of $\sum_k \frac{\partial \mathbf{y}_i(t)}{\partial \mathbf{y}_k(t-\delta t)}$ involve all the nodes connected to the i-th system, including the latter, and

$$\frac{\partial \mathbf{y}_i(t)}{\partial \mathbf{y}_k(t-\delta t)} = \frac{\partial \mathbf{y}_i(t)}{\partial \mathbf{h}_i(\ldots,\mathbf{y}_k^{\pi}(t),\ldots)} \frac{\partial \mathbf{h}_i(\ldots,\mathbf{y}_k^{\pi}(t),\ldots)}{\partial \mathbf{y}_k^{\pi}(t)} \frac{\partial \mathbf{y}_k^{\pi}(t)}{\partial \mathbf{y}_k(t)} \frac{\partial \mathbf{y}_k(t)}{\partial \mathbf{y}_k(t-\delta t)}, \ \forall k \neq i \tag{19}$$

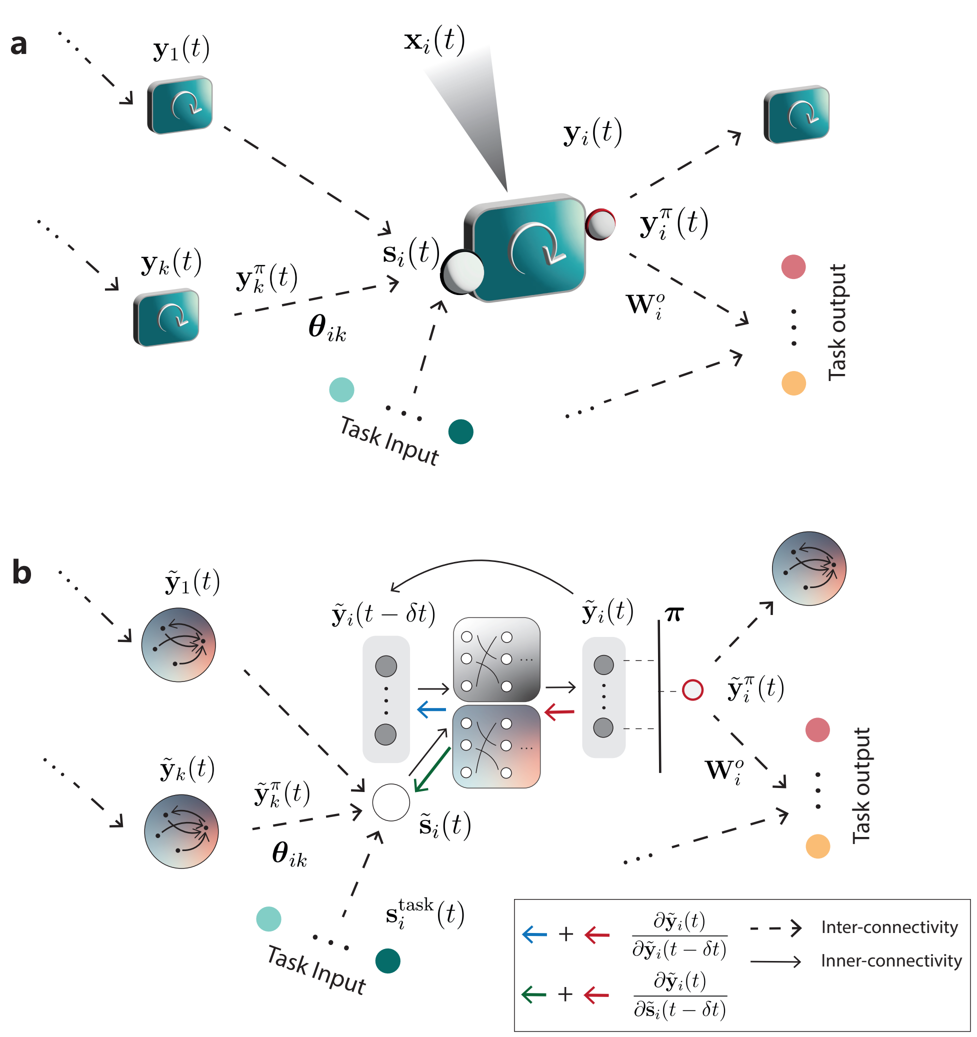

Supplementary Figure 4. Scheme of the network of experimental devices (green squares) (**a**) and of neural-SDEs (coloured circles) (**b**) illustrating the formalism adopted, where $y_i(t)$ denotes the activity at time t of node i, $s_i(t)$ the external input signal to node i which is made up of a weighted sum of parameters connecting node k in a previous layer $\theta_{i,k}$, and the variable controlling information exchange $y_k^{\pi}(t)$. $x_i(t)$ represents the latent variables describing the evolution of activity for node i. Simulated equivalents of experimental variables are denoted with a tilde above the value. The central neural-SDE of panel **b** has been expanded to show inputs and outputs, with coloured arrows highlighting the backward paths to estimate the partial derivatives with respect to past state and external input.

BPTT and the computational graph

In this section, we consider the computational graph for a feedforward network structure composed by dynamical digital twins, providing more information on the derivation of Eqs.34, 35, 36, 37 and 38 in the Methods of the Main Text. We will first recap the notation adopted. Consider hidden layers indexed by $l = 1,...,\mathrm{L}$. We denote by $\mathbf{z}_l(t) = \big(\mathbf{y}_{l,1}(t), \mathbf{y}_{l,2}(t), ...\big)$ the vector containing all states of layer l and by $\mathbf{S}_l(t) = \big(\mathbf{s}_{l,1}(t), \mathbf{s}_{l,2}(t), ...\big)$ the overall input. Analogously, we can define $\mathbf{z}_l^{\boldsymbol{\pi}}(t) = \Big[\boldsymbol{\pi}\big(\mathbf{y}_{l,1}(t)\big), \boldsymbol{\pi}\big(\mathbf{y}_{l,2}(t)\big), ...\Big]$ for the collection of variables responsible for device communication. Panel a of Fig.5 shows the computational graph related to device inter-communication across time. At time t, the high dimensional states $\mathbf{y}_{l,i}(t)$ of an example device i in layer l are projected through $\boldsymbol{\pi}$. The collective projected activities are then captured by the $\mathbf{z}_l^{\boldsymbol{\pi}}$ variable, which is then multiplied by the appropriate connectivity matrix and fed into the subsequent layer. This communication process is repeated for each time step and layer of the network. At the same time, the digital twin are responsible to evolve the activities of the system through time (panel b of Supplementary Figure 5). Indeed, the integration of the neural-SDEs permits to pass from $\mathbf{y}_{l,i}(t)$ to $\mathbf{y}_{l,i}(t+\delta t)$, or collectively from $\mathbf{z}_l(t)$ to $\mathbf{z}_l(t+\delta t)$. The overall computation graph of the system is indeed the superposition of panel a and b of Supplementary Figure 5. We notice how the digital twins act blockwise on the activities $\mathbf{z}_l(t)$, and consequently, the Jacobians $\frac{\partial \mathbf{z}_l(t)}{\partial \mathbf{z}_l(t-\delta t)}$ and $\frac{\partial \mathbf{z}_l(t)}{\partial \mathbf{S}_l(t)}$ have a block diagonal structure as follows

$$\frac{\partial \mathbf{z}_l(t)}{\partial \mathbf{z}_l(t-\delta t)} = \begin{pmatrix} \frac{\partial \mathbf{y}_{l,1}(t)}{\partial \mathbf{y}_{l,1}(t-\delta t)} & \mathbf{0}_{\mathrm{N_y}\times\mathrm{N_y}} & \cdots & \mathbf{0}_{\mathrm{N_y}\times\mathrm{N_y}} \\ \mathbf{0}_{\mathrm{N_y}\times\mathrm{N_y}} & \frac{\partial \mathbf{y}_{l,2}(t)}{\partial \mathbf{y}_{l,2}(t-\delta t)} & \cdots & \mathbf{0}_{\mathrm{N_y}\times\mathrm{N_y}} \\ \vdots & \vdots & & \vdots \\ \mathbf{0}_{\mathrm{N_y}\times\mathrm{N_y}} & \mathbf{0}_{\mathrm{N_y}\times\mathrm{N_y}} & \cdots & \frac{\partial \mathbf{y}_{l,\mathrm{M}_l}(t)}{\partial \mathbf{y}_{l,\mathrm{M}_l}(t-\delta t)} \end{pmatrix}$$

$$\frac{\partial \mathbf{z}_l(t)}{\partial \mathbf{S}_l(t-\delta t)} = \begin{pmatrix} \frac{\partial \mathbf{y}_{l,1}(t)}{\partial \mathbf{s}_{l,1}(t-\delta t)} & \mathbf{0}_{\mathrm{N_y}\times\mathrm{N_s}} & \cdots & \mathbf{0}_{\mathrm{N_y}\times\mathrm{N_s}} \\ \mathbf{0}_{\mathrm{N_y}\times\mathrm{N_s}} & \frac{\partial \mathbf{y}_{l,2}(t)}{\partial \mathbf{s}_{l,2}(t-\delta t)} & \cdots & \mathbf{0}_{\mathrm{N_y}\times\mathrm{N_s}} \\ \vdots & \vdots & & \vdots \\ \mathbf{0}_{\mathrm{N_y}\times\mathrm{N_s}} & \mathbf{0}_{\mathrm{N_y}\times\mathrm{N_s}} & \cdots & \frac{\partial \mathbf{y}_{l,\mathrm{M}_l}(t)}{\partial \mathbf{s}_{l,\mathrm{M}_l}(t-\delta t)} \end{pmatrix}$$

where M_l are the number of devices in layer l and $\mathrm{N_y}$ denotes the dimensionality of the device state $\mathbf{y}$. In our settings where we adopt delays to render the description of the system Markovian, $\mathrm{N_y} = \mathrm{N_x}\big(\mathrm{N}_{delay}+1\big)$ (see previous sections on neural-ODE and neural-SDE modelling). Practically speaking, this implies that we can compute the forward pass of the network independently for each device in a layer, meaning that we can accomplish this by including the different devices in the batch dimension to reduce computational time. Finally, following the dependencies depicted in Fig.5, we can derive the iterative equation for BPTT. Notice however, that we need to combine panel a and b

$$\frac{\mathrm{d}\mathscr{L}}{\mathrm{d}\mathbf{z}_l(t)} = \frac{\mathrm{d}\mathscr{L}}{\mathrm{d}\mathbf{z}_l(t+\delta t)}\frac{\partial \mathbf{z}_l(t+\delta t)}{\partial \mathbf{z}_(t)} + \frac{\mathrm{d}\mathscr{L}}{\mathrm{d}\mathbf{z}_{l+1}(t)}\frac{\partial \mathbf{z}_{l+1}(t)}{\partial \mathbf{s}_{l+1}(t)}\boldsymbol{\theta}_{l+1}^{\top}\frac{\partial \mathbf{z}_l^{\boldsymbol{\pi}}(t)}{\partial \mathbf{z}_l(t)}$$

which can be used in the context of digital twins and/or PAT by substituting appropriately the symbols $\tilde{\cdot}$ and/or $\hat{\cdot}$ as explained in Methods.

Initial conditions of physical devices

As the initialization of the activities of a dynamic system is a fundamental aspect of determining its dynamics, the considered devices need to be set to some starting initial conditions. This is particularly relevant in episodic settings, where each input sequence may require independent classification or correspond to distinct desired behaviours. Here, the devices are initialized to certain conditions at the beginning of each sequence. This is analogous to the starting hidden activities, typically set to zero, of a recurrent neural network within software models. While starting activities can be arbitrarily defined in software, experimental procedures naturally lead to a distribution of initial conditions. To accurately simulate the network of devices in software, it becomes relevant to experimentally obtain a probability distribution p_{IC} over initial conditions $\mathbf{y}_{\mathrm{IC}} = \Big(\mathbf{x}(t_0), ..., \mathbf{x}(t_0 + \mathrm{N}_{delay}\delta t)\Big)$ Subsequently, sampling is performed to establish the starting activity of each simulated device, replicating the process that would occur in experimental settings.

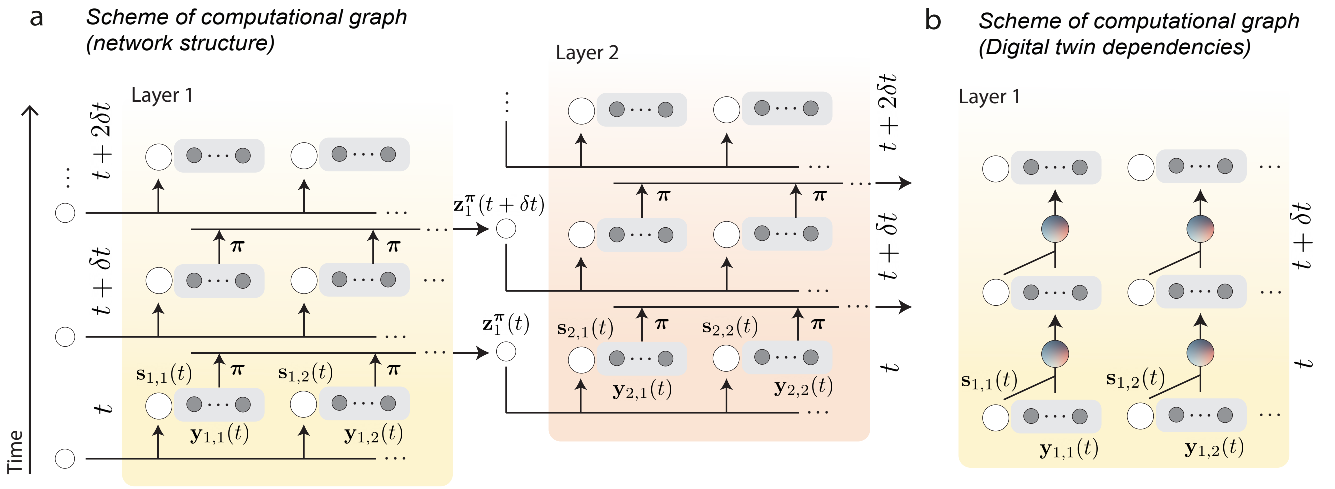

Supplementary Figure 5. Illustrative scheme of the computational graph for a feedforward network over time t where each layer is composed by dynamical devices/ digital twins. Panel **a** provides a scheme for device intercommunication between layers, where $s_{l,i}$ denotes device input and $y_{l,i}$ denotes device state for node i in layer l, and z^{π} the weighted value passed to the next layer. Panel **b** illustrates the dependencies across time captured by the digital twins. The overall computational graph is the superposition of the two panels, and backpropagating through all the dependencies will lead to BPTT for the system considered, where backpropagation is performed on the digital twins emulating the real devices with respect to input $s(t)$ and past state $y(t-\delta t)$.

Even in continuous settings, where the task can not be subdivided into independent sequences, the initial conditions still need to be set at least once. Considering devices that can be used for computational purposes and are driven by some signals, we might expect the relevance of the initial conditions to fade out at some point in time, similar to the Echo-state property[9, 10]. However, we notice that this is not true in general for dynamic systems and that the process of appropriately setting the devices' starting activities would still be important in the temporal proximity of the initial conditions.

1.3 Analytical Systems definitions

The analytical systems adopted in this work are a nonlinear leaky integrator and a Duffing oscillator, both augmented with colored noise to study the expressive ability of the neural-SDE proposed and the effectiveness of the framework in dealing with complex stochastic characteristics.

Leaky Integrator

The equation used for the leaky integrator are

$$\begin{aligned} \mathrm{d}x_1(t) &= [-\alpha_1 x_1(t) + \tanh(s(t)) + x_2(t) + x_3(t)]\,\mathrm{d}t + \sigma_1 \mathrm{dW}_1 \\ \mathrm{d}x_2(t) &= -\alpha_2 x_2(t)\mathrm{d}t + \sigma_2 \tanh(x_1(t))\,\mathrm{dW}_2 \\ \mathrm{d}x_3(t) &= -\alpha_3 x_3(t)\mathrm{d}t + \sigma_3 \tanh(s(t))\,\mathrm{dW}_3 \end{aligned} \tag{20}$$

where $\tanh(.)$ is the hyperbolic tangent, σ_i are three constants reporting the variance of the different noise sources $\mathrm{d}W_i$, α_i the leakage terms, and $s(t)$ the external signal. While the first equation defines a typical leaky integrator with a non-linear activation function, the remaining terms introduce noise over different timescales and stochastic terms that are non-linear with respect to x_1 and s. For the majority of the simulations and if not stated otherwise, we set the discretization step $\delta t = 0.1$, and the values $\alpha_1 = 1$, $\alpha_2 = 0.5$, $\alpha_3 = 1.5$, $\sigma_2 = \sigma_3 = 0.5$ and $\sigma_1 = 0.01$ and used stochastic Runge–Kutta 2 as integration method. The deterministic variant of the system is characterized exclusively by $x_1(t)$ without noise sources.

To define the datasets used to train the neural-SDE model for this system, we need to define a statistically rich ensemble of external sequences. The driving signals adopted are square functions, whose values are randomly and uniformly sampled. Mathematically, $s(t) = s_i$ for $t \in [iT_s \;\; (i+1)T_s]\ \forall i$, where T_s defines the temporal length over which the external signal remains constant, and the values of s_i are uniformly sampled in the interval $[-3 \;\; 3]$, whose range permits to sufficiently cover the regime where the hyperbolic tangent of Eq.20 varies. The training dataset for the neural-SDE comprises 1000 sequences of temporal

duration $200\delta t$, where the length T_s of the square waves is either 5 or 20. The adoption of different T_s values permits adequate observation of the temporal kernel of the system and leads to an improved neural-SDE model, but it is not strictly necessary for the results reported. In this case, we assumed observation of the variable $x_1(t)$, and we defined the neural-SDE over the variable $y(t) = x_1(t)$ without augmentation of delayed observation of x_1 (see the N-DE definition for more details). Figure 6 (left panels) shows examples of trajectories generated by these systems and the responses generated by the optimized neural-SDE.

Duffing Oscillator

The set of equations adopted for the Duffing oscillator are

$$\begin{aligned}
\mathrm{d}x_1(t) &= (x_2(t) + x_3(t) + x_4(t))\mathrm{d}t \\
\mathrm{d}x_2(t) &= \big(\alpha_1 x_1(t) + b x_1^3(t) - c x_2(t) + s(t)\cos(\omega t)\big)\mathrm{d}t + \sigma_1 \mathrm{dW}_1 \\
\mathrm{d}x_3(t) &= -\alpha_2 x_3(t)\mathrm{d}t + \sigma_2 \tanh(x_1(t))\mathrm{dW}_2 \\
\mathrm{d}x_4(t) &= -\alpha_3 x_4(t)\mathrm{d}t + \sigma_3 \tanh(s(t))\mathrm{dW}_3
\end{aligned}$$

where the external signal is convolved through a sinusoidal function and takes the place of the parameter γ, used as a multiplicative factor of the sinusoidal function defining the Duffing oscillator. The stochasticity in the system has been introduced analogously to the non-linear leaky integrator defined above. We set $\alpha_1 = 1$, $\alpha_2 = 0.5$, $\alpha_3 = 1.5$, $\sigma_2 = \sigma_3 = 0.05$ and $\sigma_1 = 0.01$ and adopted a discretization step $\delta t = 0.005$ and stochastic Runge–Kutta 4 as integration method. Analogously to the leaky integrator, the driving external signal comprises square waves, where specific values of $s(t)$ are repeated for multiple time steps, i.e. $s(t) = s_i$ for $t \in [iT_s \quad (i+1)T_s]\ \forall i$. This allows the system to exhibit the characteristic behaviours of the Duffing oscillator while subjected to a temporally varying external signal. Thus, the setting implies that the operational timescale of the system is faster than the driving stimulus. The chosen values of T_s defining the datasets of sequences are 20 and 50, while $s_i \in [-0.2 \quad 0.2]$, over which the modelled Duffing oscillator can bifurcate in proximity of $s_i = \{-0.1, 0, 0.1\}$. As a consequence, the system will exhibit bifurcations that might be triggered by stochasticity.

We assume observation of the system position $x_1(t)$ and velocities $x_2(t)$, but not of the other stochastic variables. The input representation of the neural-SDE is $\mathbf{y}(t) = \Big(x_1(t), x_2(t), x_1(t-\delta t), x_2(t-\delta t), x_1(t-2\delta t), x_2(t-2\delta t)\Big)$, where we notice $\mathrm{N}_{delay} = 2$. The training dataset comprises 2000 sequences of temporal length $200\delta t$. Supplementary Figure 6 compares trajectories sampled from the distribution of the stochastic variants of the leaky integrator (left panels) and Duffing oscillator (right) with the trajectories generated by the neural-SDE. In each panel, the systems have been subjected to 100 repetitions of identical input sequences. The average of the neural-SDE generated (yellow lines) and reference trajectories (white circles) calculated analytically are in excellent agreement. Additionally, the distributions of dynamics generated by the model and analytically are equivalent through visual inspection, showing the ability of the model to replicate both deterministic and stochastic behaviour of the target system. The neural-SDE model can consequently capture various statistical properties of the target system, despite partial observation of the variables defining the systems in question. The stochastic Duffing oscillator can exhibit bifurcations at various instants with low probability ($< 1\%$, bottom right panel). Crucially, the neural-SDE can approximately reproduce the rare event, the dynamics after the bifurcation, and its associated probability. To accurately capture this behaviour, the inclusion of delayed observations of the system activities proved to be necessary.

1.4 Analysis of Cascade Learning

Cascade learning and isolating error propagation

Cascade learning approaches performed using experimentally gathered data as input removes any error between device/simulation mismatch in previous layers by using real data in the forward pass. First, we consider the true experimental mismatch error, $E_m(\mathbf{o}_{network})$, between experimental network output, $\mathbf{o}$, and the model network output $\hat{\mathbf{o}}$, for a single hidden layer:

$$E_m(\mathbf{o}^{(\mathbf{l})}{}_{network}) = \frac{1}{n}\sum_{k=1}^{n} |o_k - \hat{o}_k|, \tag{21}$$

where n is the number of neurons in the output layer, and l is the number of hidden layers. This error will affect parameter updates via the backpropagation process. The neurons of the output layer are linear units, therefore the network output is then given by the weighted sum of activities in the penultimate layer. We consider nodes layerwise, where node $y_i^{(l)}$ denotes the ith node in layer l, which corresponds to the device output.

The nodes in layer 0, $y_i^{(0)}$, provide task-dependent inputs, hence there is no mismatch due to model inaccuracies. For a single hidden layer $l = 1$ nodes in the hidden layer, $y_j^{(1)}$, receive weighted inputs from the input layer, where $\theta_{ji}^{(0)}$ denotes the

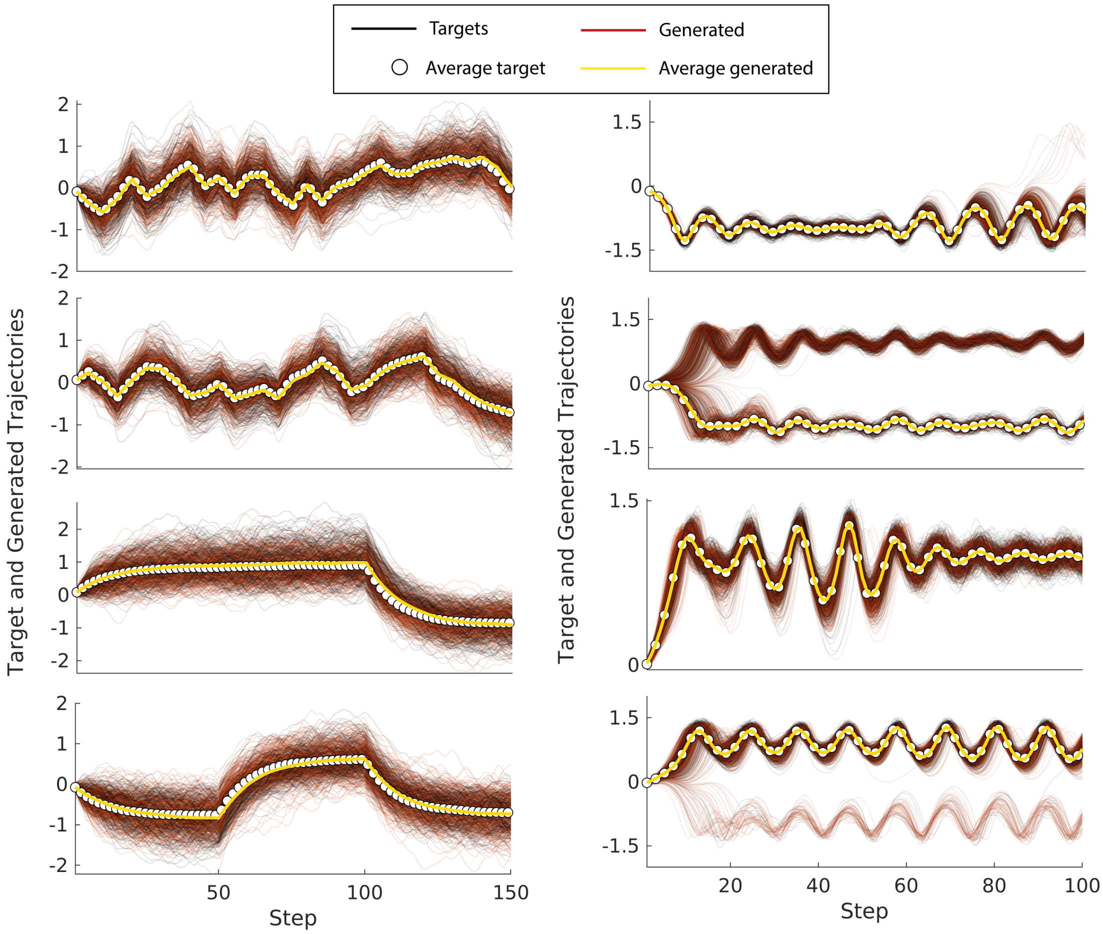

Supplementary Figure 6. Comparison between generated and target dynamics for the analytical systems considered, for the leaky integrator (left) and Duffing oscillator (right). In each panel, the systems have been driven by an external signal $\mathbf{s}(t)$, while different panels correspond to different external signals. The trajectories generated by the neural-SDE are in black, while the reference trajectories are depicted in red. In each panel, the average response is also highlighted for comparison between the model (yellow line) and the analytical system (circles), where the average is conditioned to a specific bifurcating dynamic for the Duffing oscillator. Despite the variability in the deterministic and stochastic behaviours of the systems for different external signals and the presence of bifurcations, the neural-SDE is in striking agreement with the data.

weight parameter associated with the connection between $y_i^{(0)}$ and $y_j^{(1)}$. In our framework, we use a neural-SDE to model the activity of node $y_j^{(1)}$, and $\hat{y}_j^{(1)}$ denotes the predicted output from the neural-SDE for node j in the hidden layer:

$$\hat{y}_j^{(1)} = y_j^{(1)} + \varepsilon_j^{(1)} \tag{22}$$

where ε_j denotes the mismatch between simulation and real devices for a given node. During optimization without experimental data, ε_j is unknown, and optimization instead minimizes the loss between simulated network outputs $\hat{\mathbf{o}}_{network}$. This introduces an additional mismatch error:

$$E_m(\mathbf{o}^{(\mathbf{1})}{}_{network}) = \frac{1}{n}\sum_{k=1}^{n}\left|(\sum_{j=1}^{m_1}\theta_{kj}^{(1)}\ \hat{y}_j^{(1)}) - (\sum_{j=1}^{m_1}\theta_{kj}^{(1)}\ y_j^{(1)})\right| = \frac{1}{n}\sum_{k=1}^{n}\left|\sum_{j=1}^{m_1}\theta_{kj}^{(1)}\ (\hat{y}_j^{(1)} - y_j^{(1)})\right| = \frac{1}{n}\sum_{k=1}^{n}\left|\sum_{j=1}^{m_1}\theta_{kj}^{(1)}\ \varepsilon_j^{(1)}\right| \tag{23}$$

where n denotes the number of output dimensions in the target/network output, and m_1 denotes the number of nodes in the first hidden layer. To remove this additional error, after the parameters $\boldsymbol{\theta}^{(0)}$ and $\boldsymbol{\theta}^{(1)}$ have been initially optimized to minimize the loss function of the network over the task, we then fix $\boldsymbol{\theta}^{(0)}$ and we provide all weighted inputs to nodes of experimental devices, and record activities $y_j^{(1)}$. Output weights $\boldsymbol{\theta}^{(1)}$ are then retrained on the experimental data. This removes

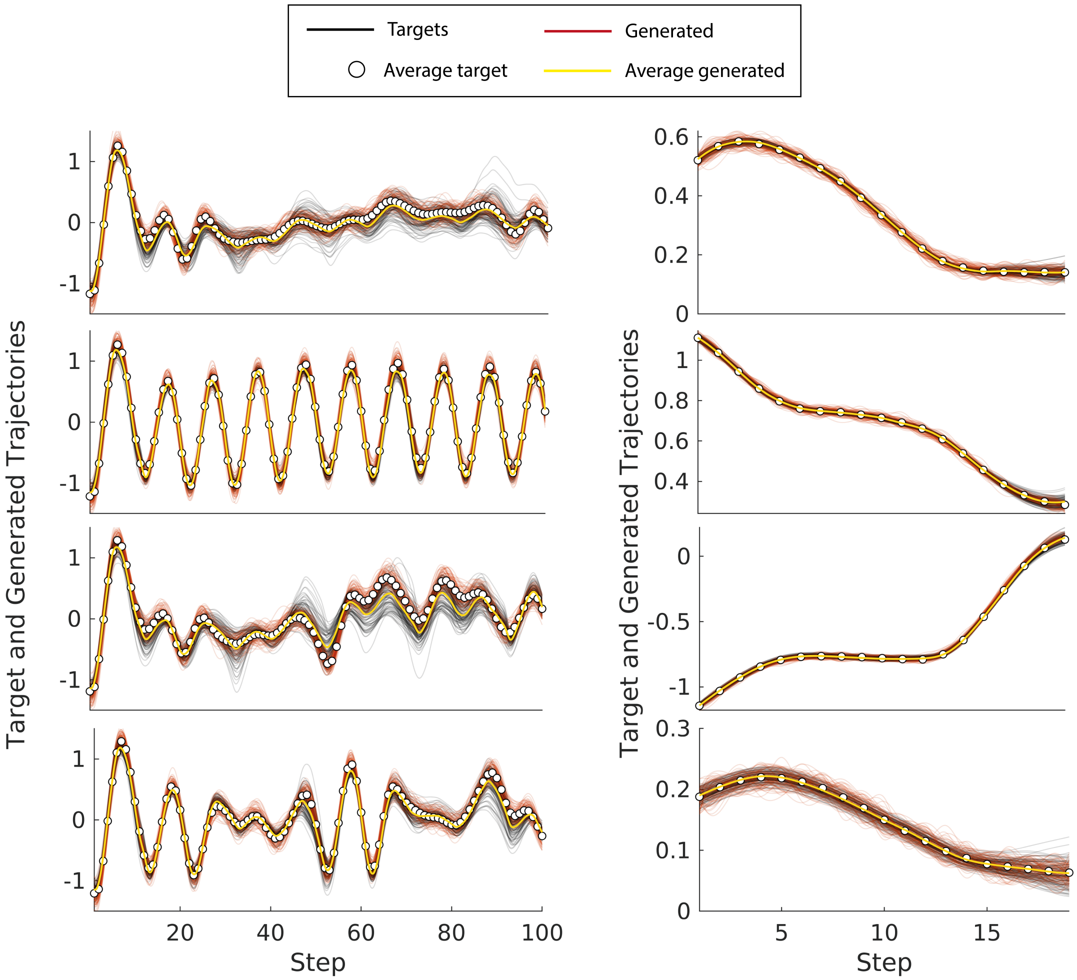

Supplementary Figure 7. Comparison between model-generated and experimentally-gathered dynamics for the physical systems considered, NRA (left) and ASVI (right). In each panel for the NRAs, the devices have been driven by an external signal $\mathbf{s}(t)$, with different panels corresponding to different external signals. Similarly to Figure 6, the trajectories generated by the neural-SDE are in black, while the experimental trajectories are depicted in red. The right panels illustrate the magnitude of specific frequencies in the high-dimensional ASVI response as time progresses for a single input sequence.

the model-experiment errors $E_m(\mathbf{o^{(l)}}_{network})$ and allows direct optimization of samples of the true experimental error $E(\mathbf{o}_{network})$.

To add a second hidden layer, we remove the output connections from the first hidden layer, $\boldsymbol{\theta}^{(1)}$, and add a new layer of nodes $\mathbf{y^{(2)}}$ and an output layer. Parameters $\boldsymbol{\theta}^{(1)}$ now describe connections between the two hidden layers, and $\boldsymbol{\theta}^{(2)}$ the connections from the new hidden layer to the output. Crucially, the input connections to the first hidden layer $\boldsymbol{\theta}^{(0)}$ remain fixed, and the experimentally gathered data for $\mathbf{y^{(1)}}$ is used for the forward pass through the first hidden layer. Hence, mismatches in neuronal activity with the physical system are only present on the nodes $\mathbf{y^{(2)}}$. The error of the network is, therefore:

$$E_m(\mathbf{o^{(2)}}_{network}) = \frac{1}{n}\sum_{k=1}^{n}\left|\sum_{j=1}^{m_2}\theta_{kj}^{(2)}\ \varepsilon_j^{(2)}\right| \tag{24}$$

This process can be recursively performed for any desired number of hidden layers. It allows for an entire network to be extensively trained while gathering only a single epoch of data for each node in the network. For l hidden layers, the error of

the output due to device vs model mismatch will be:

$$E_m(\mathbf{o}^{(\mathbf{l})}{}_{network}) = \frac{1}{n}\sum_{k=1}^{n}\left|\sum_{j=1}^{m_l}\theta_{kj}^{(l)}\ \varepsilon_j^{(l)}\right| \tag{25}$$

Cascade learning bounds the additional backpropagated error caused by the model-device mismatch for the weights $\boldsymbol{\theta}^{(l)}$ by the quantity $\delta_{device} = \sum_{j=1}^{m_l} |\theta_{kj}^{(l+1)} \varepsilon_j^{(l+1)}|$ per output node. As each previous layer is fixed before adding a new layer, any errors on the calculated partial derivatives of a node are not combined with layers ahead of it. However, it is important to notice that each hidden layer suffers this error, and the constraints added by fixing connections during the correction stage may lead to an overall suboptimal solution.

Empirical limitations of cascade learning on performance.
To compare the performance of cascade learning with fully optimized networks unaffected by simulation–reality mismatch, we repeated the Mackey–Glass time series prediction task using simulations of analytically defined leaky integrator neurons, forming a dynamical feedforward network consistent with our study. Supplementary Figure 8 presents results for predicting the first five future time steps of the input sequences used, evaluated across increasing numbers of hidden layers. For both approaches, 40,000 training iterations were performed on sequences of length 90, with backpropagation through time applied only to the final 60 inputs, and a batch size of 50 sequences.

We observe that the cascade learning approach tends to saturate earlier with respect to depth, and generally converges to slightly lower performance than full optimization. While cascade learning improved performance in our physical experiments—by isolating error propagation due to simulation–reality mismatch—in the idealized case of noiseless, perfectly modelled systems, full optimization yields superior results.

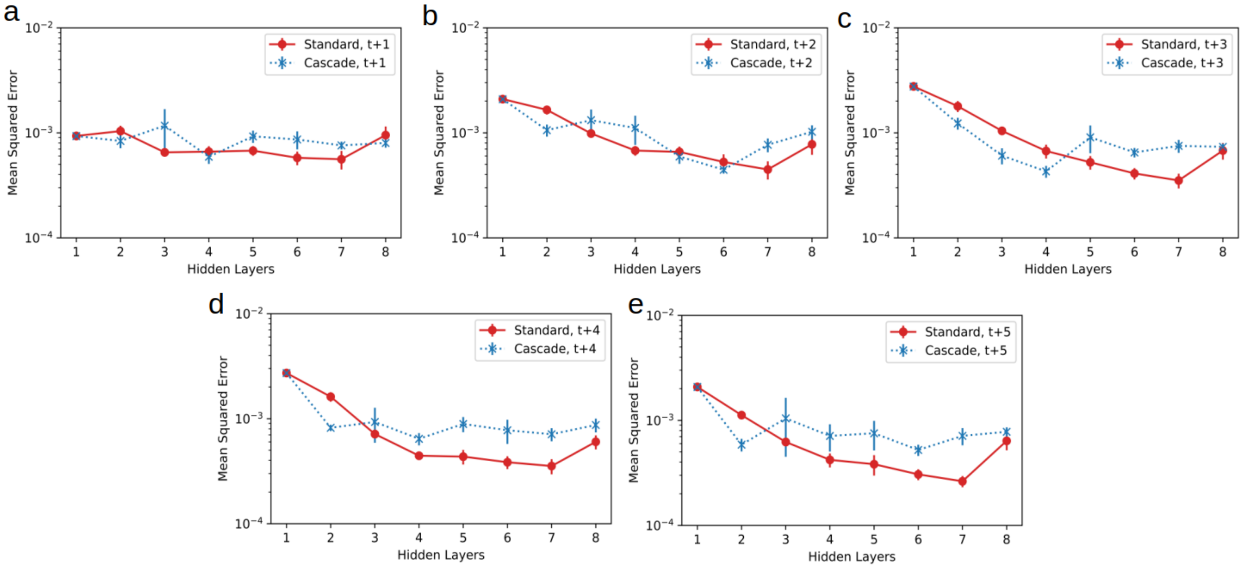

Supplementary Figure 8. A comparison between mean squared error versus number of hidden layers in the Mackey-Glass future prediction task for (a) t+1, (b) t+2, (c) t+3, (d) t+4, and (e) t+5, for networks of leaky-integrators trained via standard backpropagation through time through the full network (red circles), and layer-wise via the cascade learning algorithm (blue crosses). Each hidden layer in the network features 100 nodes. Error bars show standard deviation across 10 runs, training models independently. Training was performed for 40,000 iterations, with a batch size of 50, signal lengths of 70 inputs and excluding the first 20 steps of input from backpropagation through time.

1.5 Generalising PAT to dynamical settings

To exploit digital twins for approximating gradients of a physical system without requiring an explicit mathematical description, our framework shares strong conceptual parallels with the Physics-Aware Training scheme (PAT). In Wright et al. (2022), the authors focus on systems with static responses. They correct the behavior of their digital twins and the associated gradient

estimates by sampling real device responses to given inputs—thus incorporating the physical systems directly in the training loop during each parameter update. As discussed in the main text, our framework can effectively optimize physical networks without this continuous data sampling by adopting neural stochastic differential equations (neural-SDEs). Nevertheless, the PAT framework could still contribute valuable information to bridge the reality-simulation gap. In this section, we extend PAT to accommodate interconnected dynamical physical systems. We begin by adapting PAT to our formalism for static devices and then generalize it to the dynamical case.

Consider a network of static systems where each device i is described by the function $\mathbf{x}_i = \mathbf{f}_i\big(\mathbf{s}_i\big)$, with $\mathbf{s}_i = \sum_{j\in C} \boldsymbol{\theta}_{ij}\mathbf{x}_j$, and C being the set of devices connected to i. Using digital twins, the Jacobian can approximate this as

$$\frac{\partial \mathbf{x}_i}{\partial \mathbf{s}_i} \approx \frac{\partial \tilde{\mathbf{x}}_i}{\partial \tilde{\mathbf{s}}_i} \approx \frac{\partial \mathbf{f}_i(\tilde{\mathbf{s}}_i|\phi)}{\partial \tilde{\mathbf{s}}_i}$$

where $\mathbf{f}_i(\cdot|\phi)$ is the digital twin model and $\tilde{\mathbf{s}}_i = \sum_{j\in C} \boldsymbol{\theta}_{ij}\tilde{\mathbf{x}}_j$ is the simulated input. Under the PAT framework, we correct the simulated input $\tilde{\mathbf{s}}_i$ using the real sampled input $\mathbf{s}_i$, yielding

$$\frac{\partial \mathbf{x}_i}{\partial \mathbf{s}_i} \approx \frac{\partial \tilde{\mathbf{x}}_i}{\partial \mathbf{s}_i} \approx \frac{\partial \mathbf{f}_i(\mathbf{s}_i|\phi)}{\partial \mathbf{s}_i}$$

Although the system response is still computed through the digital twin, and therefore denoted with a tilde and by the parametrized function $\mathbf{f}(\cdot|\boldsymbol{\theta})$, the Jacobian is now evaluated at the real input. In the context of deep neural networks, where each device in a layer receives inputs from the previous layer, this correction is propagated layer by layer, and the input at a layer l is corrected by sampling the physical responses at layer $l-1$. Let $\mathbf{z}_l = \big(\mathbf{y}_{l,1}, \mathbf{y}_{l,2}, \ldots\big)$ be the responses of layer l, and $\mathbf{S}_l = \big(\mathbf{s}_{l,1}, \mathbf{s}_{l,2}, \ldots\big)$ the corresponding inputs. Then the backpropagation algorithm under PAT for static devices and an MLP with an example external input $\mathbf{s}^{task}$ becomes:

$$\frac{\partial \mathscr{L}}{\partial \boldsymbol{\theta}_{L+1}} = \frac{\partial \mathscr{L}}{\partial \mathbf{o}} \mathbf{z}_L^\top \tag{26}$$

$$\frac{\partial \mathscr{L}}{\partial \boldsymbol{\theta}_l} = \frac{\partial \mathscr{L}}{\partial \tilde{\mathbf{z}}_l} \frac{\partial \tilde{\mathbf{z}}_l}{\partial \mathbf{s}_l} \mathbf{z}_{l-1}^{\mathrm{T}}, \quad \text{for } 1 < l < L+1 \tag{27}$$

$$\frac{\partial \mathscr{L}}{\partial \boldsymbol{\theta}_1} = \frac{\partial \mathscr{L}}{\partial \tilde{\mathbf{z}}_1} \frac{\partial \tilde{\mathbf{z}}_1}{\partial \mathbf{s}_1} (\mathbf{s}^{task})^\top \tag{28}$$

$$\frac{\partial \mathscr{L}}{\partial \tilde{\mathbf{z}}_l} = \frac{\partial \mathscr{L}}{\partial \tilde{\mathbf{z}}_{l+1}} \frac{\partial \tilde{\mathbf{z}}_{l+1}}{\partial \mathbf{s}_{l+1}} \boldsymbol{\theta}_{l+1} \tag{29}$$

where $\mathscr{L}$ is the loss function and $\mathbf{o}$ is the output. The Jacobians $\dfrac{\partial \mathscr{L}}{\partial \tilde{\mathbf{z}}_l}$ remain estimated due to their reliance on digital twins in deeper layers.

To extend this to dynamical systems, we need to consider backpropagation through time (BPTT). Let $\mathbf{z}_l(t)$ be the activities at time t, $\mathbf{S}_l(t)$ the corresponding inputs and $\boldsymbol{\Pi}(\mathbf{z}_l)$ the projections defining inter-device communication. Then, BPTT is described by (see Supplementary Figure 5 for a scheme of the computational graph)

$$\frac{\mathrm{d}\mathscr{L}}{\mathrm{d}\boldsymbol{\theta}_{L+1}} = \sum_{t\leq \mathrm{T}} \frac{\partial \mathscr{L}}{\partial \mathbf{o}(t)} \frac{\partial \mathbf{o}(t)}{\partial \boldsymbol{\theta}_{L+1}} \tag{30}$$

$$\frac{\mathrm{d}\mathscr{L}}{\mathrm{d}\boldsymbol{\theta}_l} = \sum_{t\leq \mathrm{T}} \frac{\mathrm{d}\mathscr{L}}{\mathrm{d}\mathbf{z}_l(t)} \frac{\partial \mathbf{z}_l(t)}{\partial \boldsymbol{\theta}_l} = \sum_{t\leq \mathrm{T}} \frac{\mathrm{d}\mathscr{L}}{\mathrm{d}\mathbf{z}_l(t)} \frac{\partial \mathbf{z}_l(t)}{\partial \mathbf{s}_l(t)} \Big[\boldsymbol{\Pi}\big(\mathbf{z}_l(t)\big)\Big]^\top, \quad \text{for } 1 < l < L+1 \tag{31}$$

$$\frac{\mathrm{d}\mathscr{L}}{\mathrm{d}\boldsymbol{\theta}_1} = \sum_{t\leq \mathrm{T}} \frac{\mathrm{d}\mathscr{L}}{\mathrm{d}\mathbf{z}_1(t)} \frac{\partial \mathbf{z}_1(t)}{\partial \boldsymbol{\theta}_1} = \sum_{t\leq \mathrm{T}} \frac{\mathrm{d}\mathscr{L}}{\mathrm{d}\mathbf{z}_1(t)} \frac{\partial \mathbf{z}_1(t)}{\partial \mathbf{s}_1(t)} \big(\mathbf{s}^{task}(t)\big)^\top \tag{32}$$

$$\frac{\mathrm{d}\mathscr{L}}{\mathrm{d}\mathbf{z}_l(t)} = \frac{\mathrm{d}\mathscr{L}}{\mathrm{d}\mathbf{z}_l(t+\delta t)} \frac{\partial \mathbf{z}_l(t+\delta t)}{\partial \mathbf{z}_l(t)} + \frac{\mathrm{d}\mathscr{L}}{\mathrm{d}\mathbf{z}_{l+1}(t)} \frac{\partial \mathbf{z}_{l+1}(t)}{\partial \mathbf{s}_{l+1}(t)} \boldsymbol{\theta}_{l+1}^\top \frac{\partial \boldsymbol{\Pi}(\mathbf{z}_l)}{\partial \mathbf{z}_l} \tag{33}$$

Here, T denotes the final time step of the sequence under consideration. The total derivatives capture all influences on a variable, including both explicit and implicit dependencies, whereas the partial derivatives account only for direct, immediate relationships.
In contrast to the static case, BPTT features two Jacobian terms: one reflecting the dependencies of the system state with respect to its input $\frac{\partial \mathbf{z}_l(t+\delta t)}{\partial \mathbf{S}_l(t)}$, and the other of the system states across time $\frac{\partial \mathbf{z}_l(t+\delta t)}{\partial \mathbf{z}_l(t)}$. These equations assume true (unobserved) system dynamics. In practice, we estimated all terms via digital twinning and neural-SDE, and this would lead to simply adopting $\tilde{}$ across all variables but the external input. With PAT, we can sample real device states over time to better estimate Jacobians. However, sampling at every integration step is costly and requires access to high-dimensional internal states $\mathbf{y}(t)$, which may be inaccesible and/or experimentally expensive. To address this, we propose sampling at a lower frequency ω_s, correcting the digital twin periodically while using simulation in between. The modified BPTT equations under PAT with sampled updates are:

$$\frac{\mathrm{d}\mathscr{L}}{\mathrm{d}\boldsymbol{\theta}_{L+1}} = \sum_{t\leq \mathrm{T}} \frac{\partial \mathscr{L}}{\partial \hat{\mathbf{o}}(t)} \frac{\partial \hat{\mathbf{o}}(t)}{\partial \boldsymbol{\theta}_{L+1}} \tag{34}$$

$$\frac{\mathrm{d}\mathscr{L}}{\mathrm{d}\boldsymbol{\theta}_l} = \sum_{t\leq \mathrm{T}} \frac{\mathrm{d}\mathscr{L}}{\mathrm{d}\tilde{\mathbf{z}}_l(t)} \frac{\partial \tilde{\mathbf{z}}_l(t)}{\partial \boldsymbol{\theta}_l} = \sum_{t\leq \mathrm{T}} \frac{\mathrm{d}\mathscr{L}}{\mathrm{d}\tilde{\mathbf{z}}_l(t)} \frac{\partial \tilde{\mathbf{z}}_l(t)}{\partial \hat{\mathbf{s}}_l(t)} \Big[\boldsymbol{\Pi}\big(\hat{\mathbf{z}}_l(t)\big)\Big]^\top, \quad \text{for } 1 < l < L+1 \tag{35}$$

$$\frac{\mathrm{d}\mathscr{L}}{\mathrm{d}\boldsymbol{\theta}_1} = \sum_{t\leq \mathrm{T}} \frac{\mathrm{d}\mathscr{L}}{\mathrm{d}\tilde{\mathbf{z}}_1(t)} \frac{\partial \tilde{\mathbf{z}}_1(t)}{\partial \boldsymbol{\theta}_1} = \sum_{t\leq \mathrm{T}} \frac{\mathrm{d}\mathscr{L}}{\mathrm{d}\tilde{\mathbf{z}}_1(t)} \frac{\partial \tilde{\mathbf{z}}_1(t)}{\partial \hat{\mathbf{s}}_1(t)} \big(\mathbf{s}^{task}(t)\big)^\top \tag{36}$$

$$\frac{\mathrm{d}\mathscr{L}}{\mathrm{d}\tilde{\mathbf{z}}_l(t)} = \frac{\mathrm{d}\mathscr{L}}{\mathrm{d}\tilde{\mathbf{z}}_l(t+\delta t)} \frac{\partial \tilde{\mathbf{z}}_l(t+\delta t)}{\partial \hat{\mathbf{z}}_(t)} + \frac{\mathrm{d}\mathscr{L}}{\mathrm{d}\tilde{\mathbf{z}}_{l+1}(t)} \frac{\partial \tilde{\mathbf{z}}_{l+1}(t)}{\partial \hat{\mathbf{s}}_{l+1}(t)} \boldsymbol{\theta}_{l+1}^\top \frac{\partial \boldsymbol{\Pi}\big(\hat{\mathbf{z}}_l(t)\big)}{\partial \hat{\mathbf{z}}_l(t)} \tag{37}$$

$$\hat{\mathbf{z}}(t) = \mathbf{z}(t)\delta\big(t\%\omega_f\big) + \tilde{\mathbf{z}}(t)\,\big(1 - \delta\big(t\%\omega_f\big)\big) \tag{38}$$

where Eq.38 defines the sampling process. Here, δ denotes the Dirac function, % the modulo operator, and $\hat{}$ the quantities periodically corrected by the experimental data. We omitted the definitions of $\hat{\mathbf{o}}(t)$, $\hat{\mathbf{s}}(t)$ for simplicity, but these are analogous to Eq.38. We observe that the $\hat{}$ quantities appear primarily in the partial derivatives' denominators and in the second terms of the outer products, as they represent outputs that can be corrected. In contrast, the total derivatives, which propagate these estimates over time, inherits the $\tilde{}$ notation to indicate their dependence on subsequent times and layers. As in the static case, the Jacobians exhibit a block-diagonal structure. These modified equations, comprising Eqs.34, 35, 36, 37 and 38 define Algorithm 2, which extends PAT to networks of dynamical systems.

Comparison with the PAT framework

As outlined above, we extended the PAT framework to dynamical systems by correcting both the Jacobian terms and the forward responses of the devices using activity traces sampled from the real systems, as summarized in Algorithm 2. Implementing PAT directly on physical hardware used here would be experimentally demanding due to the intensive measurement requirements and device characteristics. We have estimated the training time using the neural-ODE and PAT in Supplementary Figure 9 across a range of data acquisition rates. Instead, we establish a reference baseline using analytically defined models. In this case, correcting the digital twin responses corresponds to sampling the true system states obtained through numerical integration of the governing differential equations.

As highlighted above, applying these corrections at every integration step is experimentally expensive. To address this, we also explore alternative sampling strategies. Specifically, we evaluate performance under three regimes: (1) correction at every integration step (i.e., maximum frequency), (2) correction every 10 integration steps, and (3) a single correction at the end of the sequence. While the final strategy may appear overly coarse, it remains relevant—particularly for classification tasks, where decisions are typically made at the final time step.

In all these strategies, PAT is applied to neural-ODE models rather than neural-SDEs. This choice allows us to reduce the reality-simulation gap without requiring the model itself to capture system stochasticity, as the stochastic behaviour is instead reflected directly in the sampled responses from the real system. Backpropagation Through Time (BPTT) is thus performed on the corrected neural-ODEs. Importantly, we focus on the stochastic variants of the analytical systems, as the digital twins based on neural-ODEs were already capable of successfully training the network in the absence of explicit noise modelling.

The resulting performance, after transferring the optimized weights to the true analytical systems, is shown in red and orange bars in Supplementary Figure 10.a, positioned between the accuracies of the neural-SDE and neural-ODE models. We

Algorithm 1: Forward pass through feedforward digital twins network

Considering an input sequence $\mathbf{s}^{task}(t_0), \mathbf{s}^{task}(t_0+\delta t), ..., \mathbf{s}^{task}(\mathrm{T})$

for $t = t_0,\ ...,\ T$ *in parallel* **do**

  $\mathbf{S}_1(t) = \boldsymbol{\theta}_1 \mathbf{s}^{task}(t)$

end

for $l = 1, ..., L$ **do**

  Initialize each device $\mathbf{y}_j$ in layer l from an experimentally gather distribution over initial conditions $p\big(\mathbf{y}(t_0)\big)$

  for $t = t_0,\ ...,\ T$ **do**

    Compute $\mathbf{z}_l(t)$ by solving in parallel for all device j in layer l

    $$\mathbf{y}_j(t+\delta t) = \mathbf{y}_j(t) + \int_t^{t+\delta t} \mathbf{f}\Big(\mathbf{y}_j(t'), \mathbf{s}_j(t'), t' | \phi_j^f\Big) \mathrm{d}t' + \int_t^{t+\delta t} \mathbf{g}\Big(\mathbf{y}_j(t'), \mathbf{s}_j(t'), t' | \phi_j^g\Big) \mathrm{d}\mathbf{W}$$

  end

  for $t = t_0,\ ...,\ T$ *in parallel* **do**

    $\mathbf{z}_l^{\pi}(t) = \mathbf{\Pi}_l \mathbf{z}_l(t)$

    if $l{<}L$ **then**

      $\mathbf{S}_{l+1}(t) = \boldsymbol{\theta}_l \mathbf{z}_l^{\pi}(t)$

    else

      $\mathbf{o}(t) = \boldsymbol{\theta}^o \mathbf{z}_L^{\pi}(t)$

    end

  end

end

Algorithm 2: Backward pass through digital twins network with PAT

Considering an input sequence $\mathbf{s}^{task}(t_0), \mathbf{s}^{task}(t_0+\delta t), ..., \mathbf{s}^{task}(\mathrm{T})$ and a loss function $\mathscr{L}$

Perform backward pass through the network structure at $t=\mathrm{T}$ to initialize all $\frac{\mathrm{d}\mathscr{L}}{\mathrm{d}\tilde{\mathbf{z}}_l}$ and gradients with respect to parameters $\Delta\boldsymbol{\theta}_l$

$$\Delta\boldsymbol{\theta}_{L+1} = \frac{\partial \mathscr{L}}{\partial \hat{\mathbf{o}}\,(\mathrm{T})}\left[\boldsymbol{\Pi}\big(\hat{\mathbf{z}}_L(\mathrm{T})\big)\right]^\top$$

$$\frac{\mathrm{d}\mathscr{L}}{\mathrm{d}\tilde{\mathbf{z}}_L} = \frac{\partial \mathscr{L}}{\partial \hat{\mathbf{o}}_L(\mathrm{T})}\boldsymbol{\theta}_L^\top$$

for $l = L-1, ..., 1$ **do**

$$\frac{\mathrm{d}\mathscr{L}}{\mathrm{d}\tilde{\mathbf{z}}_l} = \frac{\mathrm{d}\mathscr{L}}{\mathrm{d}\tilde{\mathbf{z}}_{l+1}}\frac{\partial \tilde{\mathbf{z}}_{l+1}(\mathrm{T})}{\partial \hat{\mathbf{s}}_{l+1}(\mathrm{T})}\boldsymbol{\theta}_{l+1}^\top \frac{\partial \boldsymbol{\Pi}\big(\hat{\mathbf{z}}_l(\mathrm{T})\big)}{\partial \hat{\mathbf{z}}_l(\mathrm{T})}$$

end

for $l = L, ..., 2$ **do**

$$\Delta\boldsymbol{\theta}_l = \frac{\mathrm{d}\mathscr{L}}{\mathrm{d}\tilde{\mathbf{z}}_l}\frac{\partial \tilde{\mathbf{z}}_l(t)}{\partial \hat{\mathbf{s}}_l(t)}\left[\boldsymbol{\Pi}\big(\hat{\mathbf{z}}_{l-1}(t)\big)\right]^\top$$

end

$$\Delta\boldsymbol{\theta}_1 = \frac{\mathrm{d}\mathscr{L}}{\mathrm{d}\tilde{\mathbf{z}}_1}\frac{\partial \tilde{\mathbf{z}}_1(t)}{\partial \hat{\mathbf{s}}_1(t)}\big(\mathbf{s}^{task}(t)\big)^\top$$

Perform backward pass through the network structure and time, recursively updating dependencies $\frac{\mathrm{d}\mathscr{L}}{\mathrm{d}\tilde{\mathbf{z}}_l}$ and $\Delta\boldsymbol{\theta}_l$

for $t = \mathrm{T}-\delta t, ..., t_0$ **do**

$$\Delta\boldsymbol{\theta}_{L+1} = \Delta\boldsymbol{\theta}_{L+1} + \frac{\partial \mathscr{L}}{\partial \hat{\mathbf{o}}\,(\mathrm{t})}\left[\boldsymbol{\Pi}\big(\hat{\mathbf{z}}_L(\mathrm{t})\big)\right]^\top$$

$$\frac{\mathrm{d}\mathscr{L}}{\mathrm{d}\tilde{\mathbf{z}}_L} = \frac{\mathrm{d}\mathscr{L}}{\mathrm{d}\tilde{\mathbf{z}}_L}\frac{\partial \tilde{\mathbf{z}}_l(t+\delta t)}{\partial \hat{\mathbf{z}}_l(t)} + \frac{\partial \mathscr{L}}{\partial \hat{\mathbf{o}}_L(\mathrm{T})}\theta_L^\top$$

for $l = L-1, ..., 1$ **do**

$$\frac{\mathrm{d}\mathscr{L}}{\mathrm{d}\tilde{\mathbf{z}}_l} = \frac{\mathrm{d}\mathscr{L}}{\mathrm{d}\tilde{\mathbf{z}}_L}\frac{\partial \tilde{\mathbf{z}}_l(t+\delta t)}{\partial \hat{\mathbf{z}}_l(t)} + \frac{\mathrm{d}\mathscr{L}}{\mathrm{d}\tilde{\mathbf{z}}_{l+1}}\frac{\partial \tilde{\mathbf{z}}_{l+1}(\mathrm{T})}{\partial \hat{\mathbf{s}}_{l+1}(\mathrm{T})}\boldsymbol{\theta}_{l+1}^\top \frac{\partial \boldsymbol{\Pi}\big(\hat{\mathbf{z}}_l(\mathrm{T})\big)}{\partial \hat{\mathbf{z}}_l(\mathrm{T})}$$

end

for $l = L, ..., 2$ **do**

$$\Delta\boldsymbol{\theta}_l = \Delta\boldsymbol{\theta}_l + \frac{\mathrm{d}\mathscr{L}}{\mathrm{d}\tilde{\mathbf{z}}_l}\frac{\partial \tilde{\mathbf{z}}_l(t)}{\partial \hat{\mathbf{s}}_l(t)}\left[\boldsymbol{\Pi}\big(\hat{\mathbf{z}}_{l-1}(t)\big)\right]^\top$$

end

$$\Delta\boldsymbol{\theta}_1 = \Delta\boldsymbol{\theta}_1 + \frac{\mathrm{d}\mathscr{L}}{\mathrm{d}\tilde{\mathbf{z}}_1}\frac{\partial \tilde{\mathbf{z}}_1(t)}{\partial \hat{\mathbf{s}}_1(t)}\big(\mathbf{s}^{task}(t)\big)^\top$$

end

Update parameters through optimizer of choice over all $\Delta\boldsymbol{\theta}_l$

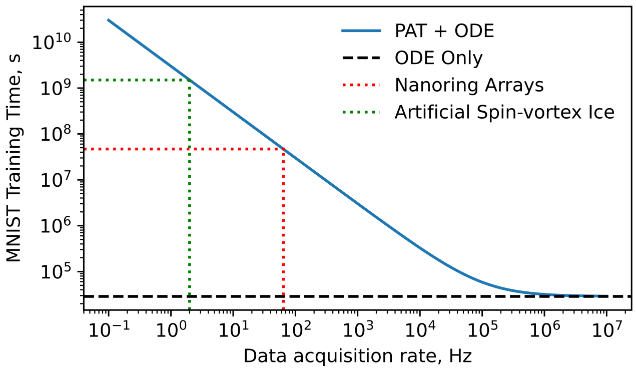

Supplementary Figure 9. Comparison of training times for a dynamic physical neural network (PNN) using the Neural-ODE methodology with PATs, versus training on the ODE alone, as a function of sampling speed for experimental devices. Vertical lines indicate the sampling rates achievable by ASVIs (2 Hz) and NRAs (64 Hz). Quoted times correspond to 30,000 iterations with a batch size of 50 and a signal length of 10 inputs, for a network comprising 200 nodes—requiring a total of 3 billion samples. The blue line is calculated by multiplying the number of samples by the acquisition time, accounting also for computation time. As simulation is independent of experimental throughput (though the computational time is dependent on model complexity; time estimates here are for an SDE model of NRA devices, and total simulation time stands at just under 8 hours for network optimization), it represents the lower bound on training duration in both methodologies.

first examine the case of the stochastic leaky integrator. Perhaps unsurprisingly, applying PAT at the highest sampling frequency achieves performance comparable to the neural-SDE models, which themselves operate at this level without any additional real-system sampling. As the sampling frequency decreases performance degrades with the lowest performance observed when correction occurs only at the end of the sequence, even though classification takes place at that final time step. This result highlights that classification relies not solely on the final outputs but on the entire dynamic evolution that leads to them. It shows that the digital description of the temporal process is critical for effective computation and optimization.

Turning to the performance of the stochastic Duffing oscillator, we observe that PAT fails to effectively train the network, even when corrections are applied at the highest sampling frequency. This limitation stems from the inherent complexity of the system's dynamics, which are sensitive to noise realizations. Specifically, the system can bifurcate between oscillatory states centred around positive or negative values depending on stochastic fluctuations (see Supp. Fig.6). This indicates a non-trivial interaction between deterministic dynamics and stochastic influences, making the system's behaviour highly variable and difficult to model accurately. In such cases, having a model that is inherently aware of the underlying stochastic processes—and capable of accounting for them during backpropagation—proves essential. While this scenario may seem extreme, it offers important insight into the limitations of deterministic models, even when augmented with correction strategies like PAT.

Finally, we note that PAT could be extended to work in conjunction with neural-SDEs, potentially further reducing the reality-simulation gap. However, given the already strong alignment between neural-SDE models and performance on the true systems, our initial attempts at integrating PAT into neural-SDEs yielded results comparable to using neural-SDEs alone.

To further illustrate how model inaccuracies can lead to poor transfer performance, particularly due to a failure to capture system stochasticity, we present in panel b of Supp. Fig.10 the performance of two neural-ODE models trained using PAT with low sampling frequencies. The panel shows test set accuracies as a function of training time, both for the neural-ODE predictions (larger circles, dashed lines) and after transferring the weights to the real system (smaller dots, solid lines). The black curve represents a case where a systematic and persistent discrepancy exists between the predicted performance of the neural-ODE and the actual performance of the true system. This gap remains largely stable throughout training. The result was obtained using a leaky integrator digital twin with correction applied once every ten integration steps. Despite this mismatch, the digital twin is still able to train the system to a reasonable performance level.

In contrast, the red curve illustrates a scenario where transfer performance is particularly unreliable. In this case, the weights optimization process exploits features of the model that do not generalize to the real system. As training progresses, this misalignment leads to overfitting: performance on the physical system initially improves but then degrades, a behaviour

characteristic of high model complexity relative to the amount of training data. Notably, this behaviour arises here due to the model relying on an inaccurate representation of the system dynamics. These results were obtained under a regime where corrections were applied only at the end of each sequence, rendering the neural-ODE effectively unaware of the noise dynamics that influence the final classification decision. This result is particularly important in understanding why using a neural-ODE as the digital twin of our physical systems can lead to degraded performance with further training (see Supp. Fig. 11).

Finally, panel c of Supp. Fig. 10 illustrates how an inappropriate sampling strategy can result in a flawed representation of the system's stochastic behaviour. In this example, the same input is presented to the neural-ODE digital twin 100 times, with a correction applied only once at the midpoint of the sequence. As observed, the neural-ODE behaves deterministically during the initial portion of the sequence, and then abruptly exhibits a distribution of responses at the sampling point. Following the correction, the system reverts to a deterministic trajectory. In this extreme, but illustrative, case, the noise signature is effectively lost, and the output distribution quickly collapses back to its average deterministic path.

1.6 Baseline Performance

This section presents additional results for the systems and tasks evaluated in the study.

1.6.1 Partially Observable MNIST Task: Neural ODE and Neural SDE

We report baseline results for the partially observable MNIST task (see Figure 3, main text) using networks composed of deterministic variants of leaky integrator and Duffing oscillator neurons. In these experiments, network connectivity was optimized using the Neural ODE framework.

Supplementary Figure 12 (panels a–d) shows performance surfaces for leaky integrator networks as a function of network size and the fraction of observable pixels. Panels a and c display the predicted accuracy from the digital twin models; panels b and d show the corresponding performance of the reference networks after transferring the optimized connectivity. In the absence of stochasticity and given the simplicity of the leaky integrator dynamics, the agreement between digital twins and reference networks is remarkably high.

We observe a clear trade-off between memory and nonlinearity that limits the performance of single-layer networks. Since the task requires both memory of past inputs and nonlinear processing, a single layer is insufficient to perform both functions effectively. In contrast, networks with two hidden layers consistently outperform their single-layer counterparts across a range of sizes. This improvement arises because the first layer can function as a memory buffer, while the second layer can implement the required nonlinearity—resulting in significantly higher classification accuracy.

Supplementary Figure 10(b) compares the performance of networks trained with Neural ODEs, Neural SDEs, and PAT across both leaky integrator and Duffing oscillator models, in deterministic and stochastic settings, using two-layer architectures. We focus here on the Neural ODE and Neural SDE results; a separate section discusses PAT-based training. As shown previously in Supplementary Figure 12, Neural ODE digital twins closely match the performance of their deterministic counterparts. For the Duffing oscillator, inputs were constrained to the range $[-0.1, 0.1]$ to avoid bifurcations during training. However, when these networks are applied to stochastic systems, performance degrades significantly (Supplementary Figure 10a, right). In contrast, networks trained using Neural SDEs retain high accuracy when transferred to their stochastic physical counterparts. This improved transferability is attributed to the noise-aware training process, which enables the digital twin to capture the system's stochastic dynamics. In this case, noise was added as described in the main text, and the input range was extended to $[-0.2, 0.2]$ to allow for noise-induced bifurcations in the Duffing oscillator's behaviour.

Extreme Learning Machine of Ring Array Networks on the MNIST benchmark

To establish a performance baseline for random connectivity structures for the partially observable MNIST task, we exploited the framework of extreme learning machines (ELM)[11]. Here, the dynamics of the nanoring array are used as a nonlinear temporal kernel, but with randomly sampled weights. The distribution of the random input weights was determined by matching the mean and variance of a Laplace distribution such that the sampled weights resemble those of a trained network, shown in Supplementary Figure 11 b. In this way, the relative magnitudes of weighted connections are comparable, but the specific weights between input channels are not maintained. This process is repeated for connectivities between additional hidden layers. The final output weights are then trained via backpropagation using a binary cross entropy loss function, identically to the digital twin framework. Since the input/hidden weights are fixed after random sampling, the dynamic properties are not considered, hence backpropagation through time is not required, similar to the reservoir computing framework[9]. The reduction in performance observed in the ELM networks highlights the importance of optimization and the consequent dependencies among specific weight values.

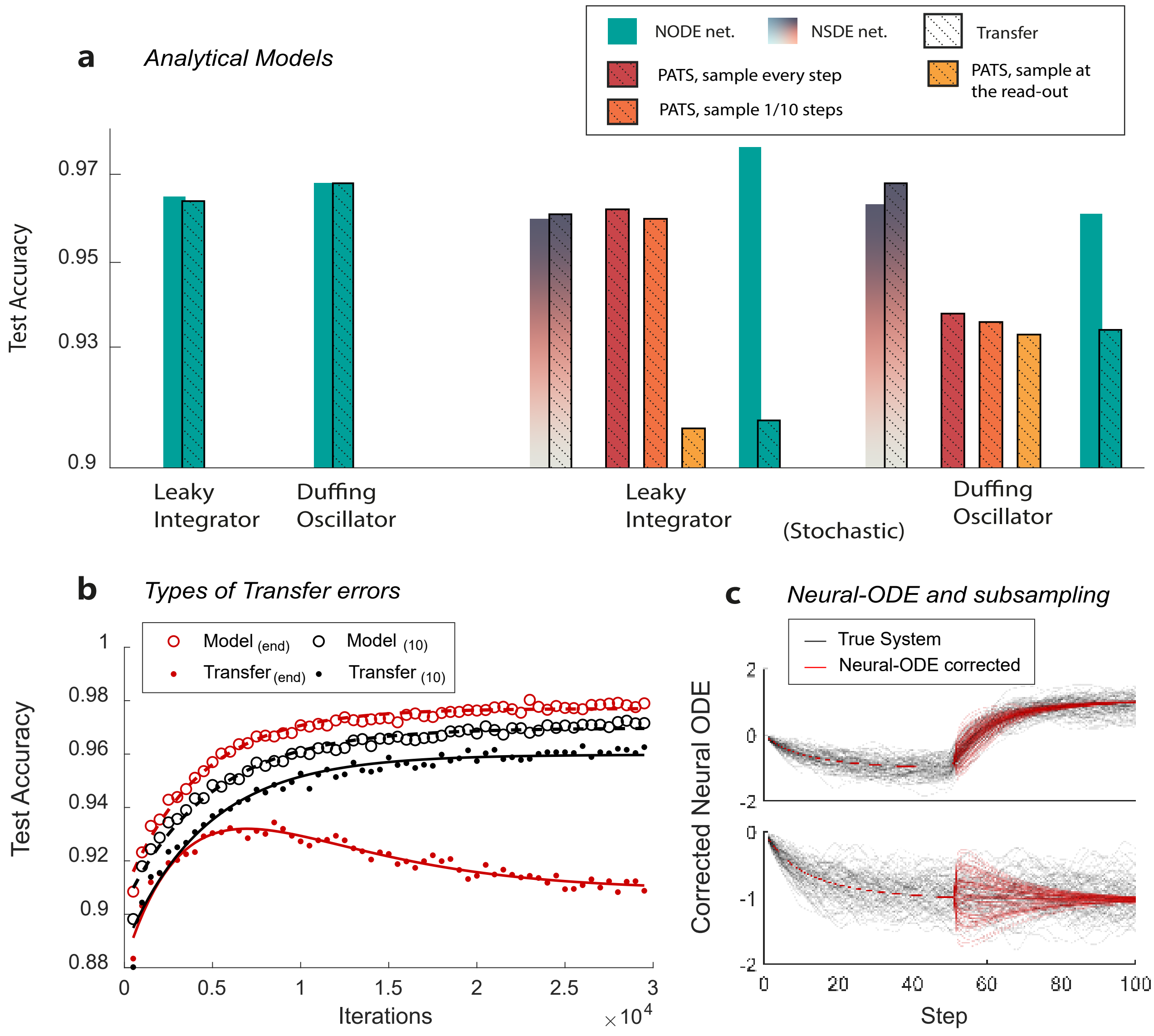

Supplementary Figure 10. **a** Reference performance on the MNIST variation task (20% visibility) for two-layer networks based on analytical models.Neural-SDEs accurately capture system dynamics in both systems and yield optimized parameters robust to stochasticity-induced bifurcations, as seen in the Duffing oscillator. In contrast, neural-ODEs fail to model noisy system variants effectively. Applying the PAT framework to correct neural-ODE responses is successful for the stochastic leaky integrator, with performance strongly dependent on the sampling strategy used. However, PAT fails for the Duffing oscillator, where complex, noise-driven bifurcations occur. Without stochastic models for gradient estimation, optimization cannot avoid regimes where bifurcation dominates. **b** Illustration of the impact of PAT noise correction frequency on MNIST task transfer accuracy. "Model/Transfer (end)" refers to a neural-ODE model and corresponding transfer accuracy for the leaky integrator where PAT correction is applied only at the final time step ("read-out," when the classification decision is made). "Model/Transfer (10)" reflects performance when PAT correction is applied at every 10th integration step. The plot shows test accuracy over training time: large white circles (dashed lines) are neural-ODE model predictions, while smaller coloured dots (solid lines) are transfer results after applying trained parameters to the experimental system. "Model (10)" (high correction frequency) exhibits systematic but stable prediction error, indicating the digital twin remains effective for training. "Model (end)" (red curves) highlights pathological optimization, where the model overfits deterministic digital features that have not been sufficiently corrected, resulting in declining performance as training proceeds. **c** Example of low-frequency PAT correction applied to a neural-ODE, showing the risk of using a deterministic model under sparse correction. The panel demonstrates that, after a brief period of variability at the correction point, the model reverts to a deterministic trajectory, failing to capture ongoing stochastic behaviour. This result shows that for systems with fast or noise-driven dynamics, PAT correction must occur frequently enough to accurately simulate the true system behaviour.

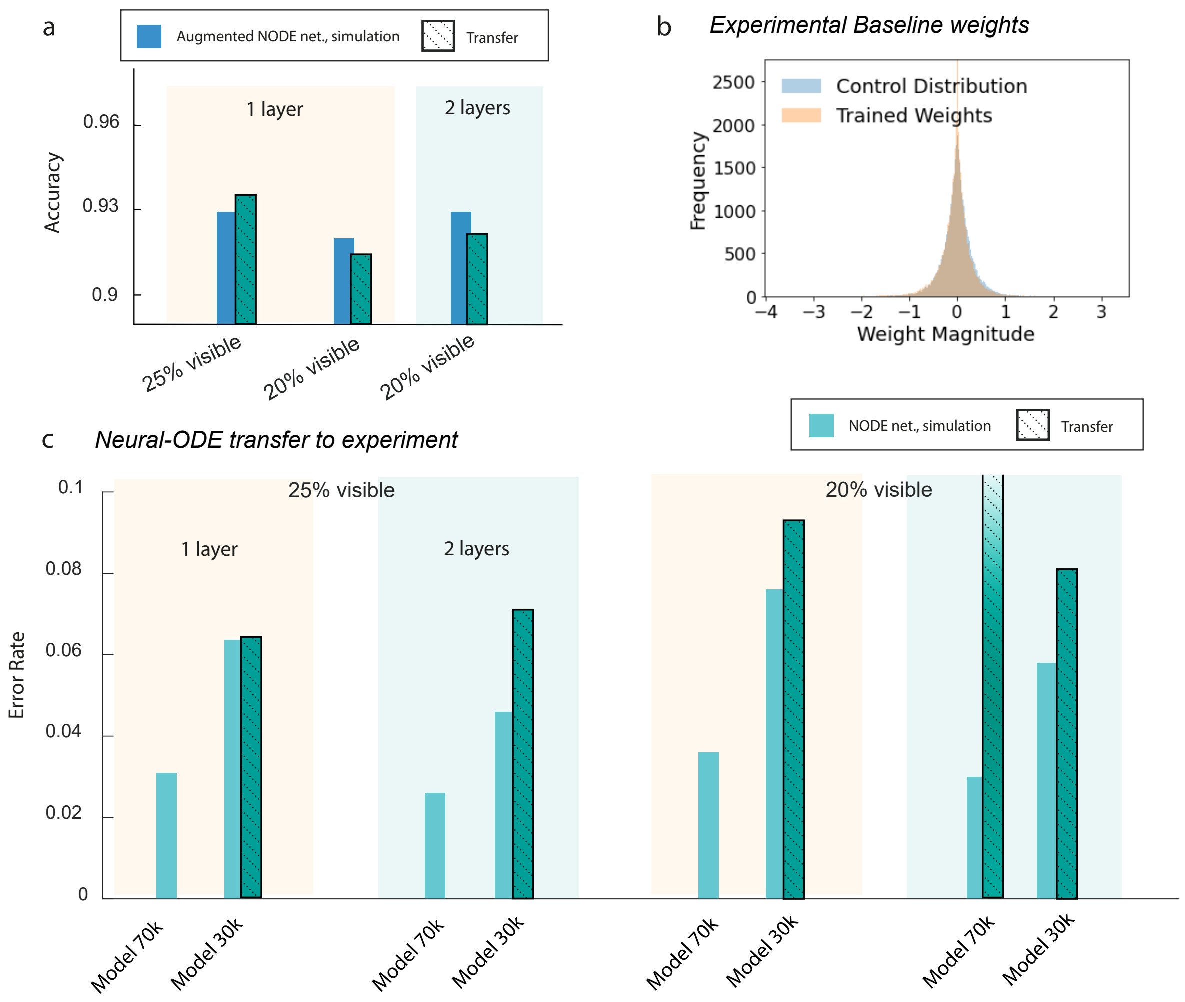

Supplementary Figure 11. **a** Performance of a Neural-ODE digital twin augmented with gaussian noise, where magnitude is modulated by the external signal and selected through statistical analysis of training data used to train the neural-SDE. The results demonstrate that simple implementations of stochasticity, and their absense from gradient calculation, means that the optimization process is unable to avoid noisy regimes, despite an improvement in transferred performance compared to neural-ODE alone. **b** Example distributions of input weights taken from (orange) a multi-layer dynamical PNN trained via the digital twin approach, and (blue) a parameter-matched Laplace distribution used for the randomly connected networks used as a baseline in MNIST tasks. **c** Performance of a network of digital twins composed by neural-ODEs in simulation and after transferring to the experiment. The models 70k and 30k refer to networks trained for 3×10^5 and 7×10^5 learning updates with a batch size of 50. While further optimization lead to improved performance in simulation, parameters transferred from the Model 70k were unable to obtain a meaningful classification. This is showcased for the two-layer network and 25% visibility. The result can be interpreted in the following way: as optimization progresses, the parameters become more dependent on the deterministic responses provided by the neural-ODE, tending to amplify the simulation-reality gap. For this reason, we had to stop the optimization process before convergence (at 3×10^5) for acceptable performance with transferred parameters. As shown in the main text, the noise-aware optimization of the neural-SDE removes this difficulty.

Extreme Learning Machine on the Neuroprosthetics Task.

Due to the inability to provide meaningful connections between hidden layers highlighted by the sharp decrease in performance with two-layer ELMs in the partially observable MNIST task, the baseline performance for the Neuroprosthetics task adopted a single-layer ELM. The width of the layer of 400 nodes has been chosen to match the two hidden layers of the optimized dynamic networks of devices. The mean and distribution of random input weights were optimized via a grid search according to the highest performance on a validation data set.

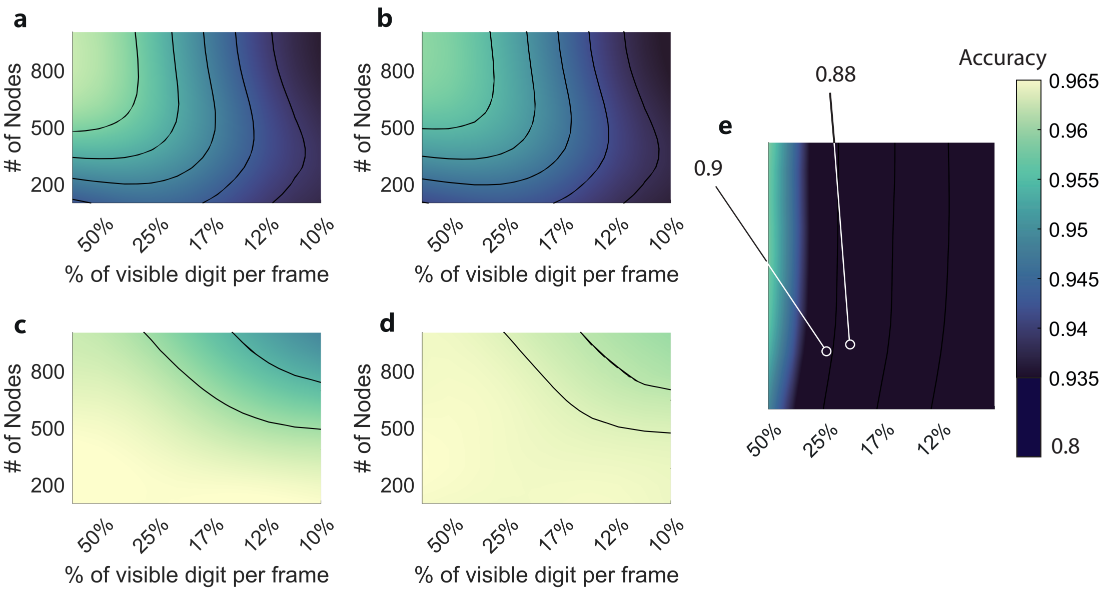

Supplementary Figure 12. Comparative performance surfaces for the partially observable MNIST task using networks of interacting analytical leaky integrators as a baseline performance for simulated dynamical neural networks using the framework. Panels **a** and **b** show results for single-layer networks, while **c** and **d** correspond to two-layer networks. The eural ODE–based digital twin models are shown in **a** and **c**; the corresponding reference networks, with transferred connectivity, are shown in **b** and **d**. Panel **e** shows the performance of a two-layer multilayer perceptron (MLP) trained on the same task. Its lower accuracy as frame visibility decreases reflects the absence of temporal memory and highlights the advantage of dynamic components in solving this task.

Similarly, we show the performance of a software MLP of identical shape to the dynamic networks and sigmoidal activation functions. To introduce a finite memory source similar to that provided by the nanoring devices, the inputs instead consisted of the previous three entries for each of the input dimensions, creating a buffer of previous inputs. Since the network itself has no dynamic properties, standard backpropagation was performed using the same binary cross-entropy loss function as in the dynamic networks.

Leaky integrators on the Neuroprosthetics Task.
Finally, we compare performance of the experimental dynamic neural networks of NRAs to software-implemented dynamic neural networks where nodes are described by analytical nonlinear leaky integrators. The software leaky-integrator networks were constructed with identical topologies as the experimental DNNs. Hyperparameter searches were conducted across the leak-rate of the underlying dynamical nodes, with leak-rates of 0.37 found to be optimal. Performance of these networks is shown in Supplementary Figure 13, and closely rivalled that of the experimental NRA networks, with the NRAs edging accuracy over the training window and thereafter, though the leaky-integrator networks had slightly higher performance on earlier timesteps.

In all cases, training was performed over the same window resembling meaningful information as was used in Figure 3. upplementary Figure 13 shows performances obtained when classifying at each timestep of the input signals for a single trained model optimized over the highlighted window, showing the optimized dynamic networks achieve greater classification accuracy maintained over a longer duration.

1.7 Energy Calculations for PNNs

As a baseline for the energy consumption for the dynamical neural networks in conventional hardware, we evaluate the number of floating point operations (FLOPs) per input for networks defined by analytical leaky integrators, as in the MNIST and neuroprosthetic tasks. The neuron update assumes the cheapest numerical integration method (Euler), meaning the effective equation for calculating neuron activity is defined by:

$$x_t = \tanh(\alpha \cdot s_t) - (1-\alpha)x_{t-1} \tag{39}$$

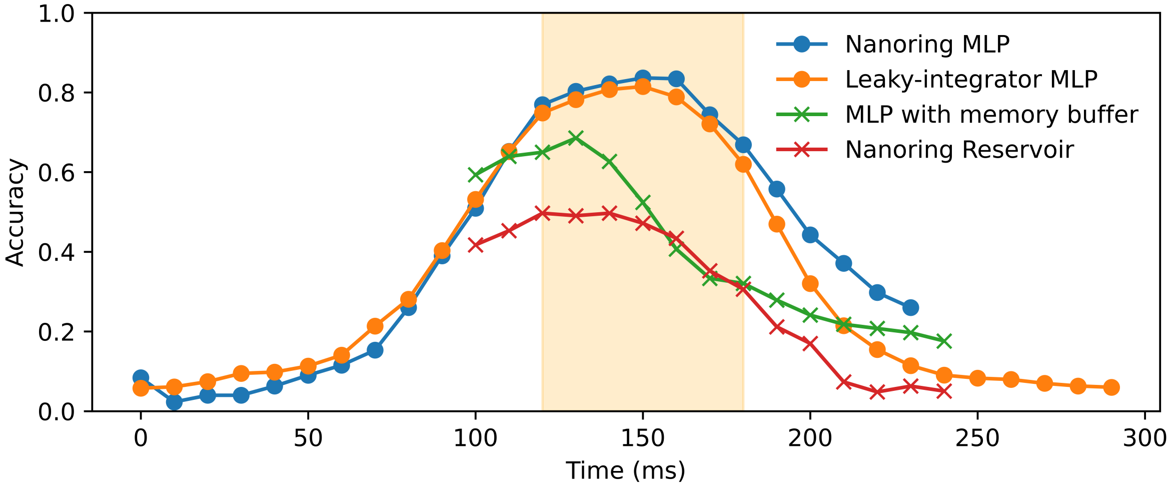

Supplementary Figure 13. Experimental and reference performance on the neuroprosthetic task as the time at which the classification decision is made varies, for optimized networks of nanoring arrays (blue), analytical leaky-integrator based networks in simulation (orange), randomly connected networks of nanoring arrays (red), and standard software neural networks with small memory buffers of the previous three inputs (green). All networks are optimized to minimize cross-entropy loss between the network prediction and movement label over a window containing the most meaningful information for classification, highlighted by the shaded orange region.

where x_t describes activity at time t, s_t the input to the neuron at time t, and α the leak rate associated with the neuron. Depending upon the optimization of the tanh operation, calculating this update equation costs between 6 and 25 FLOPs.

To calculate the cost per operation on both high-end GPU hardware (Nvidia RTX 4090), and typical computing platforms used for edge computation (Raspberry Pi 5), we take power consumption of each device (450 W for RTX 4090, 10 W for Raspberry Pi 5), and divide by the peak computational throughputs (82.6 TFLOPS for RTX4090, 31.4 GFLOPS for Raspberry Pi 5) to give an estimate for the energetic cost per floating point operation. This gives estimates for 4.45 pJ/FLOP for the RTX 4090, and 318 pJ/FLOP for the Raspberry Pi 5. Based on our earlier calculation for the number of FLOPs required per neuron update, this gives estimates for neuron updates at between **26.7pJ** and **111.25pJ** for an RTX 4090, and between **1.91nJ** and **47.75nJ** for a Raspberry Pi 5.

While the field-driven implementation for the NRAs used here has poor energy efficiency, the magnetization dynamics exploited can be driven via spin-orbit torques. Based upon simulations in MuMax3[12], the current density required to drive domain walls similarly to magnetic fields is on the order of $4 \times 10^{11}\,\mathrm{A/m^2}$ with bias fields of 0.3 T, and can achieve clock speeds of around 100 MHz. For 25x25 ring arrays as used here, with 0.5 μm diameter rings shown to have equivalent behaviours[13], this equates to 31.25 mW to drive the array. Encoding an input into a single rotation as used here, a single input costs around **313pJ**.

Based upon these calculations, the energy efficiency of the NRAs sits between high-end devices optimized for machine learning (RTX 4090), and low-power devices capable of edge computing (Raspberry Pi 5). While this proves favourable for the edge applications these devices are most appropriate for, we would like to reiterate that the focus of this paper is on the methodology employed rather than the proposal of physical devices themselves.

Recent studies into dynamic physical neural networks using spin-torque nano-oscillators (STNOs), widely considered the current state-of-the-art in spintronic computing, show suitability for dynamic PNNs in simulation, with energy estimates of around 100 fJ per neuron operation[14]. This makes STNOs a promising candidate for employing our methodology to realise these networks in hardware, with dramatic improvements in energy efficiency even compared to GPUs.

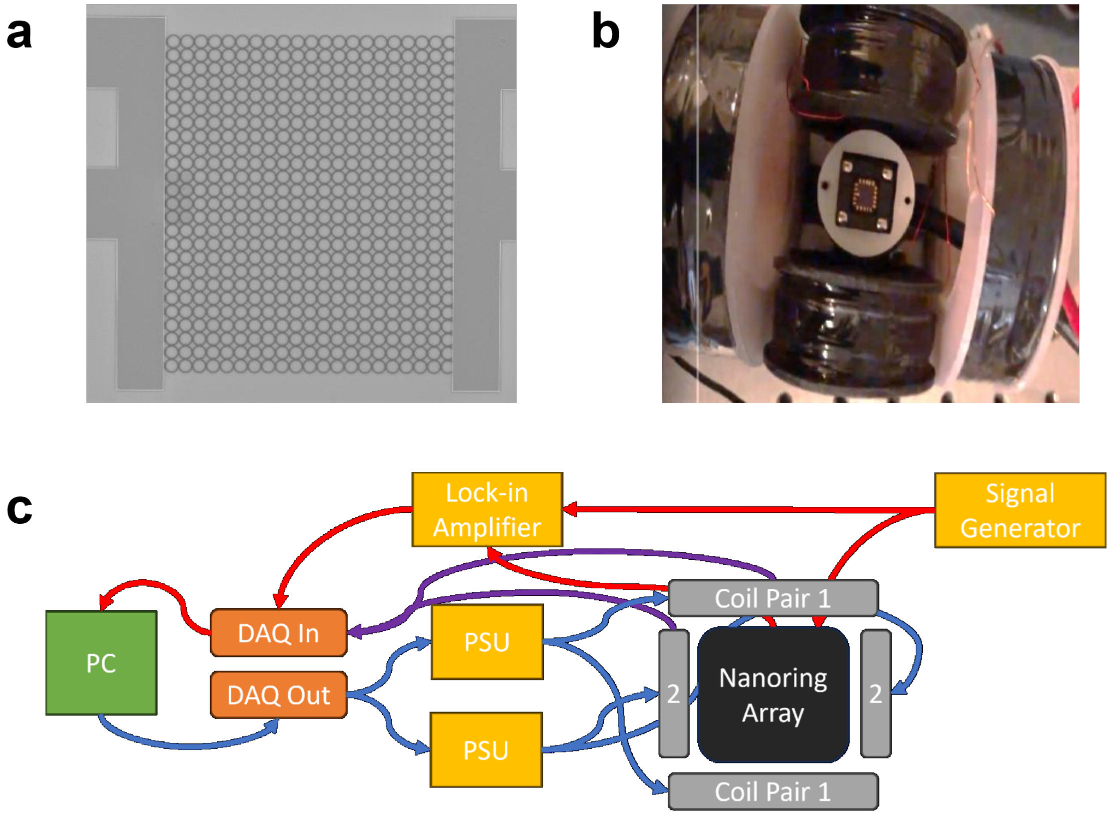

Supplementary Figure 14. **a** Scanning electron micrograph of nanoring array, showing electrical contacts patterened on top of the nanoring structure. **b** Image of the sample shown in **a** connected electrically via a chip carrier to an external current source used for anisotropic magnetoresistance measurements, mounted between custom-built electromagnet coils which provide external input signals of rotating magnetic fields to drive dynamic behaviours. **c** Schematic diagram for the experimental measurement of magnetic nanoring arrays. A desktop PC (green) interfaces between the analogue input and output functionality of a National Instruments data acquisition card (orange), which sends output voltage signals to a pair of voltage-controlled power supply units, which in turn provide rotating magnetic fields via two pairs of electromagnets (grey) uniformly over the magnetic nanoring array (black). A signal generator provides a uniform oscillating current signal to both the nanoring array, where the signal is modulated via anisotropic magnetoresistance effects, and also to a lock-in amplifier as a reference signal to filter out experimental noise. The filtered signal from the lock-in amplifier is passed to the analogue input of the data acquisition card which is then logged on the PC.

References

1. Werbos, P. J. Backpropagation through time: what it does and how to do it. *Proc. IEEE* **78**, 1550–1560 (1990).
2. Bellec, G. *et al.* A solution to the learning dilemma for recurrent networks of spiking neurons. *Nat. communications* **11**, 3625 (2020).
3. Bellec, G. *et al.* Biologically inspired alternatives to backpropagation through time for learning in recurrent neural nets. *arXiv preprint arXiv:1901.09049* (2019).
4. Chen, X. *et al.* Forecasting the outcome of spintronic experiments with neural ordinary differential equations. *Nat. communications* **13**, 1016 (2022).
5. Chen, R. T., Rubanova, Y., Bettencourt, J. & Duvenaud, D. K. Neural ordinary differential equations. *Adv. neural information processing systems* **31** (2018).
6. Kidger, P., Foster, J., Li, X. & Lyons, T. J. Neural sdes as infinite-dimensional gans. In *International conference on machine learning*, 5453–5463 (PMLR, 2021).

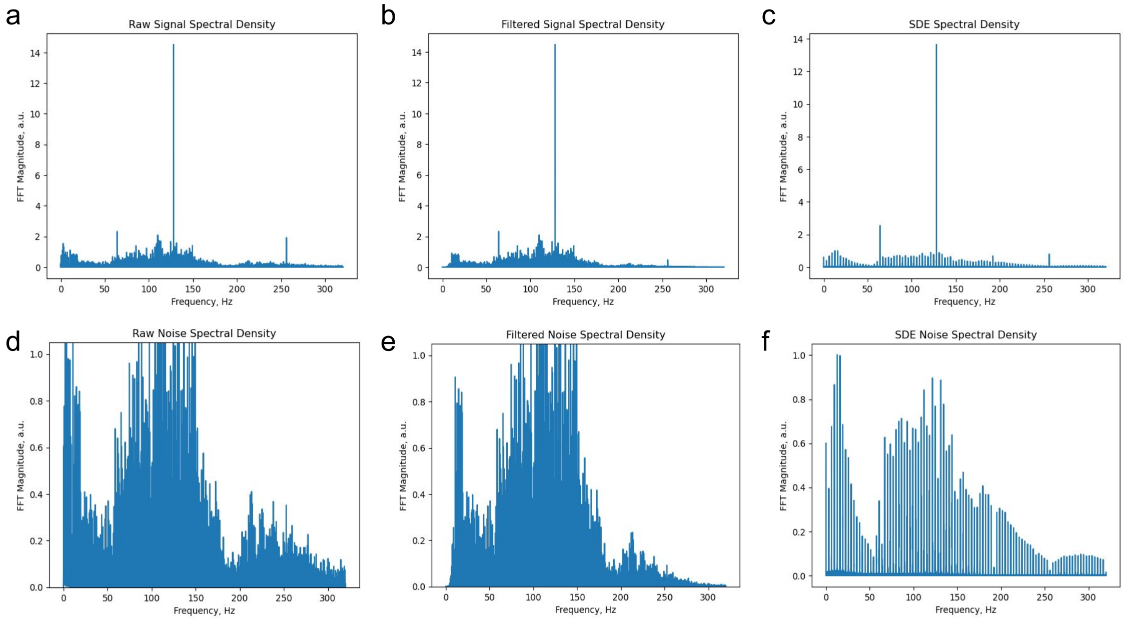

Supplementary Figure 15. (a) Power spectral density plot of raw signal gathered from nanomagnetic ring arrays. Noise mainly centres around the frequency of the main component of the AMR signal (128 Hz) as well as some noise at lower frequencies (<50 Hz) (b) Resulting spectral density of experimental data after filtering with a third-order band-pass filter with corner frequencies of 50 and 200 Hz. Reduces the scale of low frequency noise, and noise beyond 200 Hz. (c) Power spectral density of the neural-SDE prediction of input waveforms used for panels (a) and (b). While the mapping is not perfect, the SDE model is able to reproduce qualitatively the key features of the plots in (a) and (b). Panels (d), (e), and (f) focus upon the lower magnitude frequency responses largely attributed to noise of the signals plotted in panels above.

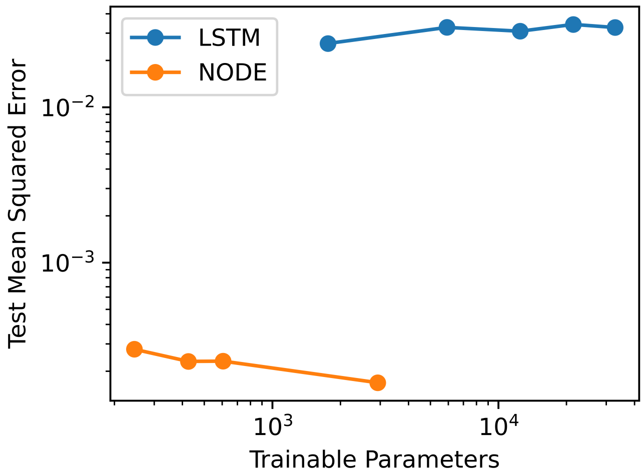

Supplementary Figure 16. A comparison between achieved mean-squared error when using different modelling paradigms to model the response of the nanoring arrays and predict unseen input/output relationships for LSTM networks using the standard package within PyTorch, and the Neural-ODE approach. It can be seen that the ODEs vastly outperform LSTMs in both terms of accuracy achieved, as well as parameter count. Training was performed across 50,000 iterations with a batch size of 50 and a signal length of 50 inputs, excluding the first 10 steps from backpropagation through time.

7. Gulrajani, I., Ahmed, F., Arjovsky, M., Dumoulin, V. & Courville, A. C. Improved training of wasserstein gans. *Adv. neural information processing systems* **30** (2017).

8. Saxena, D. & Cao, J. Generative adversarial networks (gans) challenges, solutions, and future directions. *ACM Comput.*

ODE/SDE Training (Rings)	
Dataset (ODE)	20k patterns, length 20 (1800 train, 100 val + test)
Dataset (SDE)	2k patterns, 100 repetitions, length 20 (1800 train, 100 val + test)
Batch Size	200
Iterations	50k (ODE), then 50k (SDE)
Learning Rate	0.01
Time Horizon (BPTT)	50 discretisation steps (2.5 samples)

MNIST	
Dataset	80k images (60k train, 10k each val/test, 1k experimental transfer from test set)
Batch Size	50
Iterations	30k/70k (ODE), 70k (SDE)
Learning Rate	0.003
Time Horizon (BPTT)	Full signal (observability dependant)

Neuroprosthetics	
Dataset	27 patients, 17 gestures, 10 repetitions per gesture (8 train, 1 val, 1 test)
Batch Size	10
Iterations	50k
Learning Rate	0.003
Time Horizon (BPTT)	18 inputs (360 discretisation steps)

ODE/SDE Training (ASVI)	
Dataset	13k Samples (11k train, 1k each val/test)
Batch Size	50
Iterations	20k ODE, 10K SDE
Learning Rate	0.005
Time Horizon (BPTT)	10 Samples

Mackey-Glass (Cascade)	
Dataset	6000 samples (5k train, 500 val/test), First 1000 samples of train set repeated per additional layer for cascade recorrection.
Iterations	5k (first ASVI Layer). 1 iteration of ridge regression for final output layer, with 5k iterations for updating input weights via cascade learning.
Batch Size	100
Learning Rate	0.001
Time Horizon (BPTT)	20 Samples

Supplementary Figure 17. Details of hyperparameters selected for the optimization and training data used for the neural-ODE/SDE models, partially-observable MNIST task, neuroprosthetic movement classification task, and Mackey-Glass future prediction task.

Surv. (CSUR) **54**, 1–42 (2021).

9. Jaeger, H. The "echo state" approach to analysing and training recurrent neural networks- with an erratum note. Tech. Rep., German National Research Center for Information Technology, Bonn, Germany (2001). Publication Title: GMD Technical Report.

10. Yildiz, I. B., Jaeger, H. & Kiebel, S. J. Re-visiting the echo state property. *Neural networks* **35**, 1–9 (2012).

11. Ding, S., Xu, X. & Nie, R. Extreme learning machine and its applications. *Neural Comput. Appl.* **25**, 549–556 (2014).

12. Vansteenkiste, A. *et al.* The design and verification of MuMax3. *AIP Adv.* **4**, 107133, DOI: 10.1063/1.4899186 (2014). Publisher: American Institute of Physics.

13. Allenspach, R., Bischof, A. & Heller, R. Antidot lattices for magnetic reservoir computing. *Appl. Phys. Lett.* **125** (2024).

14. Plouet, E., Sanz-Hernández, D., Vecchiola, A., Grollier, J. & Mizrahi, F. Training a multilayer dynamical spintronic network with standard machine-learning tools to perform time-series classification. *Phys. Rev. Appl.* **23**, 034051 (2025).
